# Nuclear receptor NURR1 functions to promote stemness and epithelial-mesenchymal transition in prostate cancer via its targeting of Wnt/β-catenin signaling pathway

**DOI:** 10.1038/s41419-024-06621-w

**Published:** 2024-03-26

**Authors:** Xingxing Zhang, Haolong Li, Yuliang Wang, Hui Zhao, Zhu Wang, Franky Leung Chan

**Affiliations:** 1 School of Biomedical Sciences, Faculty of Medicine, The Chinese University of Hong Kong, Hong Kong, China; 2 Department of Urology, The People’s Hospital of Longhua, Shenzhen, 518109 Guangdong, China

**Keywords:** Cancer stem cells, Prostate cancer, Transcriptional regulatory elements

## Abstract

Dysregulated activation of Wnt/β-catenin signaling pathway is a frequent or common event during advanced progression of multiple cancers. With this signaling activation, it enhances their tumorigenic growth and facilitates metastasis and therapy resistance. Advances show that this signaling pathway can play dual regulatory roles in the control of cellular processes epithelial-mesenchymal transition (EMT) and cancer stemness in cancer progression. Aberrant activation of Wnt/β-catenin signaling pathway is shown to be common in prostate cancer and also castration-resistant prostate cancer (CRPC). However, the transcriptional regulators of this pathway in prostate cancer are still not well characterized. NURR1 (*NR4A2*) is an orphan nuclear receptor and plays an important role in the development of dopaminergic neurons. Previously, we have shown that NURR1 exhibits an upregulation in isolated prostate cancer stem-like cells (PCSCs) and a xenograft model of CRPC. In this study, we further confirmed that NURR1 exhibited an upregulation in prostate cancer and also enhanced expression in prostate cancer cell lines. Functional and molecular analyses showed that NURR1 could act to promote both in vitro (cancer stemness and EMT) and also in vivo oncogenic growth of prostate cancer cells (metastasis and castration resistance) via its direct transactivation of *CTNNB1* (β-catenin) and activation of β-catenin to mediate the activation of Wnt/β-catenin signaling pathway. Moreover, we also demonstrated that NURR1 activity in prostate cancer cells could be modulated by small molecules, implicating that NURR1 could be a potential therapeutic target for advanced prostate cancer management.

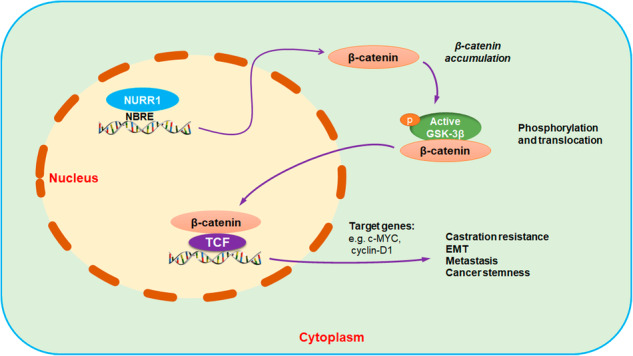

## Introduction

A significant amount of evidence indicates that a small subset of cancer cells within malignant tumors, designated as cancer stem cells or stem-like cells (CSCs), contribute significantly to tumor relapse, metastasis and therapy resistance in advanced cancer development [1, 2]. Prostate cancer stem cells (PCSCs) can be isolated from various preclinical sources and clinical tumor tissues using different methods [3, 4]. Experimental evidence suggests that PCSCs play crucial roles in therapy-resistance, disease relapse, and metastasis [5–7]. During advanced malignant growth, some cancer cells acquire with an embryonic or dedifferentiation cellular process, epithelial-mesenchymal transition (EMT), to enhance their invasion and metastasis capacity. Recent studies show that advanced prostate cancer cells with EMT phenotype exhibit certain cancer stem-like growth features (e.g. enhanced anchorage-independence and tumorigenicity), share common signaling pathways (e.g. Wnt/β-catenin, Notch, hedgehog) and express similar expression patterns (e.g. increased expression of CSC-associated transcription factors and membrane markers) [8–10]. However, the transcriptional control in linking between EMT and cancer stemness in prostate cancer is still not well understood.

Wnt/β-catenin signaling is a main signal transduction pathway in the regulation of embryonic development and maintenance of stem cells, and also normal differentiation in adult tissues [11]. It is well characterized that its aberrant expression and mutations of pathway proteins can contribute to the development of multiple cancers, exerting multiple oncogenic functions that include maintenance of cancer stem cells and tumor initiation, metastasis and anti-tumor immunity [12]. Advances show that Wnt/β-catenin signaling can play dual regulatory roles in the crosstalk between cancer stemness and EMT in cancer progression [10, 13, 14]. Aberrant activation of Wnt/β-catenin signaling and mutations of pathway members are common in prostate cancer, particularly in advanced metastatic castration-resistant prostate cancer (CRPC). However, signaling pathways or regulators mediating the activation of this pathway in prostate cancer are not well characterized. Expression analyses of prostate cancer tissues show that abnormal cytoplasmic or nuclear β-catenin expression is correlated with Gleason score and hormone-refractory status [15, 16]. Increased expressions of Wnt ligands, including Wnt-5a and Wnt-11, are shown in primary prostate cancer tissues and bone-metastasis lesions, and elevated Wnt-11 expression is correlated with PSA levels and implicated in neuroendocrine (NE) transdifferentiation [17, 18]. With this oncogenic activation, it promotes cancer stemness, activated stroma and altered microenvironment, EMT and metastasis, reciprocal crosstalk with androgen receptor (AR) and therapy-resistance in different stages of prostate cancer progression [19–23]. By whole-exome and transcriptome sequencing, somatic mutations of genes in Wnt/β-catenin pathway (including *CTNNB1*) and deletions or reduced expressions of negative regulators of Wnt/β-catenin signaling as identified in metastatic CRPC patients are predictive to acquired resistance to novel first-line hormone therapies (Abiraterone and Enzalutamide) [24, 25]. Studies in transgenic mouse models show that Wnt-5a can act as an activator of AR-mediated prostate cancer growth and Wnt-5a-mediated activation of β-catenin signaling can cooperate with Pten loss to drive AR-independent CRPC [18, 26]. However, the upstream regulation of Wnt/β-catenin signaling in cancer is still not fully characterized.

Nuclear receptor related-1 protein (NURR1, Nurr1, *NR4A2*) belongs to the NR4A subfamily of orphan nuclear receptors due to their absence of physiological or natural ligands or ligand-independence. Mouse and human homologs were originally isolated from cDNA libraries derived from mouse brain and human activated T-cells respectively [27, 28]. NURR1 shows particularly high expression in central nervous system [29, 30]. Knockout study suggests that *NR4A2* is critical in the development and maintenance of dopaminergic neurons [31, 32] and its impaired function is implicated in the pathogenesis of Parkinson’s disease [33].

Abnormal expression of NURR1 is implicated in cancer growth and progression. Aberrant overexpression of NURR1 is demonstrated in multiple cancers, including gastric, cervical, pancreatic and prostate cancer; and its increased expression is correlated with poor prognosis in patients [34–38]. NURR1 overexpression is also shown in tuberous sclerosis complex-associated benign tumors [39]. Our recent expression study demonstrates that NURR1 exhibits upregulation in PCSCs and a xenograft model of CRPC [34]. A few functional analyses suggest that NURR1 can perform certain oncogenic roles, involving induction of autophagy, ERK and AKT signaling and cell-cycle regulator CDK4 [36, 38–40]. On the other hand, decreased expression of NURR1 is shown in breast cancer, with negative correlation with lymph node metastasis but with a strong correlation with prolonged relapse free survival in patients, suggesting that NURR1 may play a dichotomous role in breast cancer [41].

In the present study, we demonstrated that NURR1 could act as a key regulator in the promotion of both EMT and cancer stemness in prostate cancer through its direct transcriptional control of *CTNNB1* and also activation of β-catenin signaling. Our results also showed that targeting of NURR1 or its mediation of β-catenin signaling could have a potential therapeutic implication in the management of CRPC.

## Materials and methods

### Cell lines and cell cultures

One immortalized prostatic epithelial cell line (BPH-1) and five prostate cancer cell lines (LNCaP, VCaP, 22Rv1, DU 145 and PC-3) were used in this study. BPH-1 was provided by Dr. Simon Hayward and VCaP was provided by Dr. Kenneth Pienta, while others were obtained from ATCC (Manassas, VA). The cell lines were grown and maintained in routine culture conditions as described previously [42, 43].

### Immunohistochemistry

Immunohistochemistry of NURR1 and immunoreactivity score analysis (IRS) was performed on prostatic tissue microarray slides (MTP1101, Taibsbio, Xi’an, China) containing hyperplasia (BPH, *n* = 20) and prostate cancer (*n* = 90, Gleason-scored) tissues using a mouse monoclonal anti-NURR1 antibody (ab60149, Abcam) following by avidin-biotin-peroxidase staining and scoring procedure as described previously [43].

### Plasmid construction and lentiviral transduction

(a) Expression plasmids: pLenti-CMV-NURR1/NR4A2 (containing insert of full-length NURR1, FLAG-labeled) and pLenti-U6-NURR1-shRNA1/2 were purchased from Vigene Biosciences (Rockville, Maryland, USA) for lentiviral transduction. FLAG-NURR1 and its DNA-binding (DBD) truncated mutant NURR1-ΔDBD (generated by fusion-PCR method) were subcloned into pcDNA3.1 as pcDNA3.1-NURR1 and pcDNA3.1-NURR1-ΔDBD for transfection. Lentivirus production was performed in 293T packing cells following procedure as described previously [44]. (b) Reporter gene plasmids: Human *CTNNB1* (β-catenin) gene promoter region (−1,321 to −1), containing one NGFI-B response element (NBRE) or consensus extended hormone response element (AAATATCT; located at −1,321 to −1,314), was PCR-amplified using genomic DNA extracted from HEK293 cells and cloned into pGL4.1 [luc] (Promega) as pGL4-CTNNB1-Luc. *CTNNB1* promoter fragment carrying the NBRE with point mutations was generated by site directed-mutagenesis-PCR method and cloned into pGL4-TK-Luc as pGL4-NURR1-NBRE-mutants1/2/3 (mutant 1: AACCATCT; mutant 2: AAATTCCT; mutant 3: AAATATTG). All plasmid constructs were validated by DNA sequencing before use.

### RNA sequencing

RNA samples were extracted from NURR1/vector-transduced cells for cDNA library construction followed by paired-end sequencing using an Illumina next-generation sequencing (NGS) platform (E-GENE Co., Ltd). Sequence reads or count values on each gene were analyzed by HTSeq to determine its original expression in samples. Fragments per kilobase per million mapped fragments or reads (FPKM), based on length of gene and read counts mapped, were used to normalize the gene expression. Genes with FPKM-value > 1 are considered to be expressed in the reference transcriptome.

### Chromatin immunoprecipitation (ChIP)

ChIP assay of *CTNNB1* promoter was performed in NURR1-HEK293 transfected cells following procedures as described previously [45]. Briefly, the genomic DNA of NURR1-HEK293 transfected cells was extracted and enzyme-digested into DNA fragments, followed by immunoprecipitation with NURR1 antibody (ab60149, Abcam) and qPCR analysis. The sequence information of primer pairs used for the *CTNNB1* gene promoter is: 5′-CTCTGTCTCAAAAAAAAACAAAACATAGTTCACACT-3′ and 5′- AATTTGTCCGTATTTTGCCTTTATTGTGCA-3′.

### Gene expression and genomics analyses

(a) The expression profile of NURR1 in clinical prostate cancer was analyzed using two gene expression microarray datasets available from ONCOMINE database (https://www.oncomine.org), and its expression correlation with β-catenin (*CTNNB1*) was also analyzed using a multi-platform dataset of primary prostate cancer available from a prostate carcinoma dataset (PRAD) from TCGA (The Cancer Genome Atlas Research Network) [46]. (b) Gene Set Enrichment Analysis (GSEA) was performed using GSEA 4.1. The signaling pathways were considered significantly enriched with FDR < 0.25 and plotted.

### In vitro cell growth analyses

(1) Cell viability assay: viable cells were determined by the colorimetric cell counting kit-8 (CCK8/WST-8) assay following manufacturer’s procedures (Beyotime Biotechnology). Briefly, cells were seeded at 10^3^ cells/well in 96-well plates and cultured in normal media with FBS upon or under various experimental conditions and duration. CCK8/WST-8 reagent was added to cultured cells (100 μl/well) followed by 1–4 h incubation. The absorbance *A*_450_ was measured using a microplate reader (Perkin Elmer Victor 3 V). (2) Non-adherent 3D-culture: anchorage-independent growth capacity (stemness) of cells was determined using an agar-based non-adherent 3D-culture method following procedures as described previously [4]. Briefly, cells were suspended in serum-free DMEM/F-12 medium (GlutaMAX, Gibco) with supplements (20 ng/mL human EGF, 20 ng/mL human basic FGF, 4 µg/mL insulin, 1 × B-27 supplement, 1% KnockOut serum replacement and 1% penicillin-streptomycin) and seeded onto agar (0.9%) -coated 24-well plates (200–500 cells/well) and cultured for 1–4 weeks for formation of spheroids. Spheroids formed were photographed and scored for their numbers and sizes. (3) Matrigel-based 3D-culture. The 3D-spheroid or colony formation capacity of cells was determined using a submerged Matrigel-based 3D-culture method [47]. Single cells suspended in serum free-medium with supplements were mixed with Matrigel and plated onto 24-well plates (density 1–2.5 × 10^3^/well) on ice. The plates with cells-Matrigel mixture were placed in a humidified incubator at 37 °C for 20 min to allow Matrigel solidification, followed by addition of serum free-medium (500 μl) to the wells. The cells suspended in Matrigel were allowed to grow for 2 weeks to generate spheroids, followed by scoring of their numbers and sizes. (4) Wound healing assay. Cell migration capacity of cells was assayed following procedures as described previously [48]. Briefly, cells were grown in normal medium with FBS in 12-well plates to 80% confluence before making wound scratches with pipette tips and had the normal medium replaced with serum free-medium. Wounded areas were photo-captured at different time points (0, 24 and 48 h) and measured using ImageJ software. (5) Transwell-invasion assay. Invasion capacity of cells was assayed following previous procedures with minor modifications [47]. Briefly, cells to be evaluated were cultured in antibiotic-containing serum free-medium for 24 h before assay. Cells suspended in serum free-medium were seeded onto the upper chamber of transwell plates with insert 8-μm pore size polycarbonate membrane coated with Matrigel (10^3^/well; 24-well plate, Corning), and with the lower chamber filled with growth medium containing 12% FBS. After incubation for 24–48 h, cells attached to membrane were fixed with ice-cool methanol for 30 min, followed by staining with methanolic 0.1% crystal violet. Stained cells attached on the upper membrane surface were removed by cotton swaps, while the migrating cells on the lower membrane surface were photographed and counted under an inverted microscope.

### Molecular biology and immunoblot analyses

(1) RT-qPCR analysis. Total RNA was extracted from cultured cells and xenograft tumors using TRIzol reagent (Molecular Research Center), followed by genomic DNA elimination and reverse transcriptase-based cDNA synthesis (PrimeScript RT reagent kit with gDNA eraser, TaKaRa). A SYBR green-based qPCR assay was performed on cDNA samples using a real-time PCR system (StepOne Real-Time PCR System, Applied Biosystems) following procedures as described previously [46]. Relative mRNA expression levels of gene targets were determined by a comparative C_T_ method (2^-ΔΔCT^), with normalization to β-actin (*ACTB*). The specificities of primers were verified by melting curve assay of PCR products. All reactions were performed in triplicate. Sequence information of primers used is listed in Supplementary Table 1. (2) Immunoblot analysis. Total cellular and nuclear proteins were extracted from subconfluent cultured cells using an ice-cooled SDS-free modified RIPA lysis buffer with cocktail of protease inhibitors (Pierce Immunoprecipitation Lysis Buffer, ThermoFisher). Denatured proteins were separated by SDS-PAGE electrophoresis and transblotted onto PVDF membranes. Immunoblot signals of separated proteins were detected by an enhanced chemiluminescence method following procedures as described previously [49] using a gel imaging system (Bio-Rad ChemiDoc XRS System). Primary antibodies used and their sources are listed in Supplementary Table 2. (3) Luciferase report assay. Dual luciferase reporter assay was performed in non-prostatic HEK293 cells co-transfected with 0.05–0.5 μg expression plasmids (pcDNA3.1-NURR1/NURR1-ΔDBD), 0.2 μg reporter plasmids (pGL4-CTNNB1-Luc/CTNNB1-NBRE-mutants-Luc) and 0.01 μg control *Renilla* reporter plasmid pRL-TK using polyethylenimine (PEI) transfection reagent. Forty-eight hours post transfection, firefly and control *Renilla* luciferase activities were determined (dual luciferase reporter system kit, Vazyme Biotech) following procedures as described previously [44]. Luciferase reporter activity was normalized to the control pRL-TK activity and expressed as fold change relative to pcDNA3.1 or pGL4. All assays were repeated in independent triplicates.

### In vivo tumorigenicity assay and CRPC xenograft tumors

In vivo tumorigenicity of NURR1- and vector-transduced VCaP cells and also their responsiveness to androgen-deprivation were evaluated in intact SCID mice followed by castration at 4 weeks post inoculations following similar procedures as described previously [49]. When the castration-relapse tumors re-grew to sizes of about 0.8 cm^3^, tumor-bearing mice were treated with a Wnt production inhibitor IWP-2 (i.p. injection, 0.05 mmol/g body weight; Abmole Bioscience) [50] for a duration of 2 weeks. At the end of experiments, tumors were harvested for histology or snap-frozen in liquid nitrogen for molecular biology analyses.

### Zebrafish embryo metastasis assay

An in-house propagated transgenic zebrafish line with blood vessels expressing green fluorescent protein (GPF) (*flk1:GFP*) was used for the in vivo assay of metastasis potential of prostate cancer cells. NURR1/vector-transduced prostate cancer cells (PC-3-NURR1 and PC-3-vector) were pre-labeled with red fluorescent protein (RFP) by transfection with pRFP plasmid (Addgene plasmid #26924) [50] and suspended in PBS (1 × 10^7^ cells/ml). The suspended pRFP-labeled cells were microinjected (total 200 cells/2 nl) using a pulled borosilicate glass micropipette into the sinus venosus region of 48-h post-fertilization (hpf) tricaine-anesthetized embryos placed onto a warm injection plate. After cell injection, embryos were returned to aquaria at 28 °C for 3–5 days with aquarium water renewed every two days. At 5-day post injection, anesthetized embryos were placed onto an agarose-coated plate and imaged for metastatic cells under a fluorescence inverted microscope (Olympus IX83). For quantification analysis, distinct RFP-labeled cells in the embryos were counted or evaluated by qPCR of cells migrated in different regions. All procedures were performed with approval from the CUHK-Animal Experimentation Ethics Committee (AEEC).

### Bioinformatics and statistical analyses

Results of continuous variables were expressed as mean ± SD and statistically analyzed by unpaired Student’s *t*-test or one-way analysis of variance (ANOVA). Differences were considered significant with *P* values < 0.05.

## Results

### NURR1 exhibits an upregulation expression in prostate cancer tissues and prostate cancer cells

**Fig. 1:**
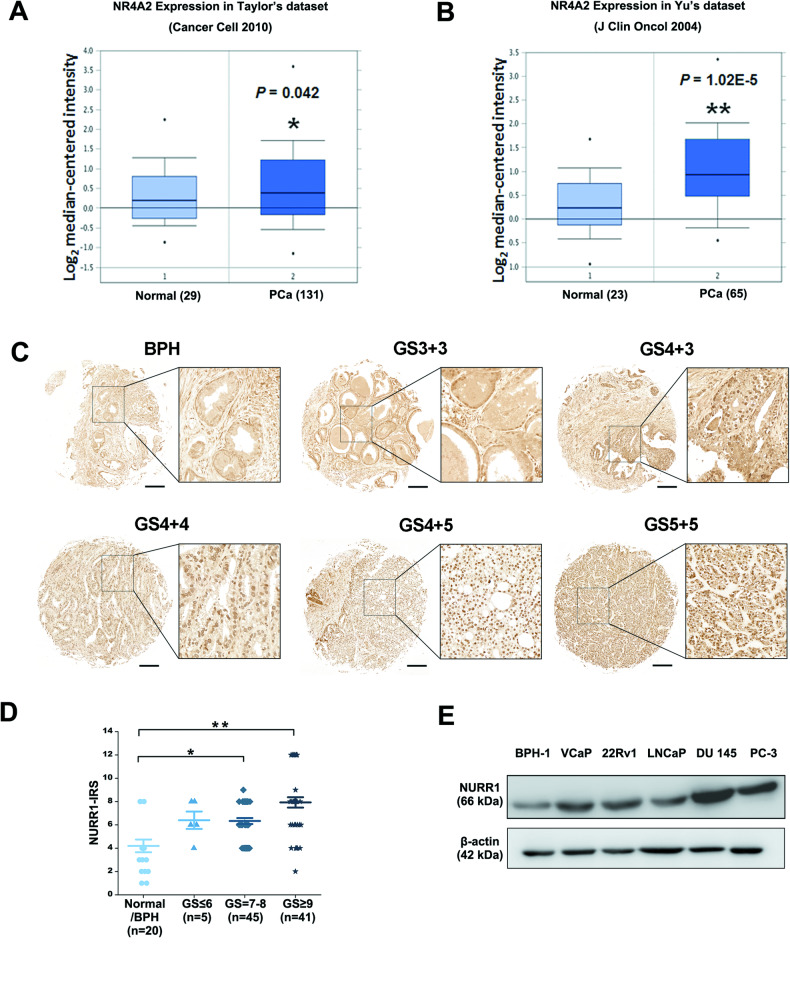
NURR1 exhibits an upregulation in clinical prostate cancer tissues and prostate cancer cell lines. **A**, **B** Transcriptome analysis of two microarray datasets of clinical prostate cancer tissues reveal that NURR1 exhibits an increased expression pattern in prostate cancer. **C**, **D** Immunohistochemical analysis of NURR1 in prostate cancer tissue microarrays. **C** Representative micrographs show the NURR1-immunostained tissue spots of BPH and prostate cancer tissues. More intense nuclear immunosignals of NURR1 were detected in cancer cells than that in glandular epithelial cells in BPH and adjacent normal tissues. Magnification, × 400; bars = 500 μm. **D** NURR1-IRS analysis. Results showed that high Gleason score (GS) prostate cancer lesions expressed significant higher NURR1 expression than in normal or BPH tissues. **E** NURR1 immunoblots. Multiple prostate cancer cell lines expressed higher levels of NURR1 than immortalized prostatic epithelial cells BPH-1. *P < 0.05; **P < 0.001 versus normal or BPH.

Previously, we have identified that NURR1 manifests a common upregulation in both the prostate cancer stem cells (PCSCs) -enriched 3D-cultured prostatospheroids derived from different prostate cancer cell lines and a CRPC xenograft model (CRPC-VCaP) based on the castration-relapse growth of VCaP cells in castrated mice, suggesting that NURR1 might perform an oncogenic role in prostate cancer [34]. Here, we further confirmed its expression pattern in clinical prostate cancer using two expression microarray datasets of prostate cancer available from ONCOMINE database [51, 52]. Results revealed that NURR1 showed higher expression levels in clinical prostate cancer tissues than in normal prostate tissues (Fig. 1A, B). Immunohistochemical tissue microarray analysis of NURR1 demonstrated that cancer cells in high Gleason-score lesions in hormone-naïve prostate cancer expressed higher nuclear immunoreactivity than that in glandular epithelial cells in non-cancerous tissues, which expressed either weak or negative immunoreactivity (Fig. 1C). Immunoreactivity score analysis (IRS) also showed that high GS lesions displayed higher NURR1 immunoreactivity than non-cancerous tissues (Fig. 1D). Moreover, immunoblot analysis also showed that multiple selected prostate cancer cell lines expressed higher protein levels of NURR1 than immortalized prostatic epithelial cells (Fig. 1E).

### Transcriptome analysis suggests NURR1 is involved in activation of Wnt/β-catenin signaling

**Fig. 2:**
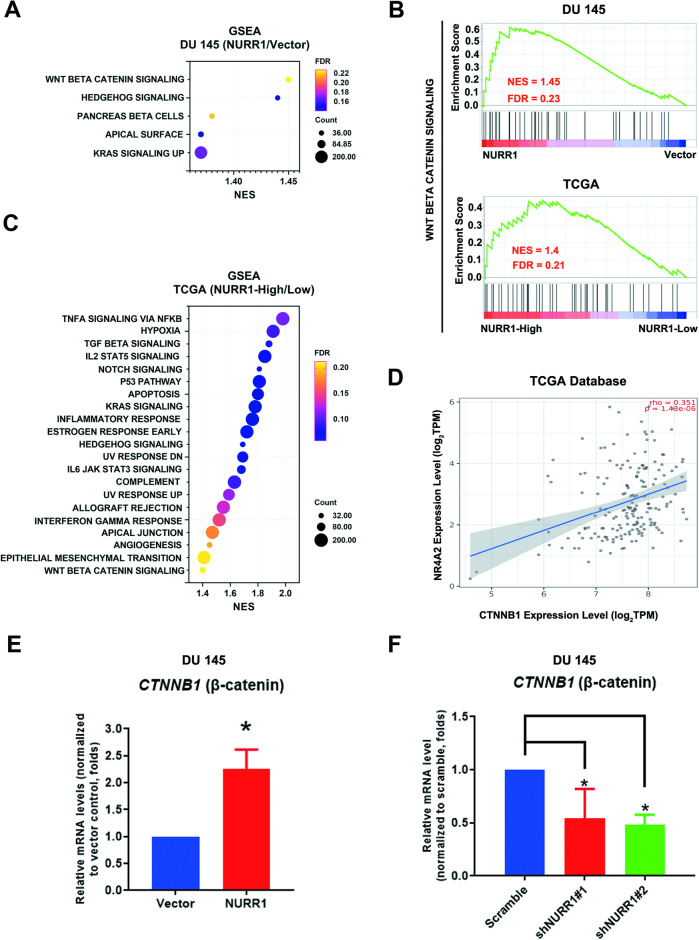
The Wnt/β-catenin signaling is involved in NURR1-overexpressed prostate cancer. **A** Bubble plot displaying Gene Set Enrichment Analysis (GSEA) of RNA-sequencing data of the DU 145-NURR1 and -Vector infectants. The result reveal that the Wnt/β-catenin signaling is the most significantly enriched pathway in NURR1-overexpression (NURR1) group compared to the control (Vector) group. **B** GSEA results of enriched Wnt/β-catenin signaling in DU 145-NURR1 and -Vector infectants, as well as in NURR1-High and -Low groups. **C** Bubble plot showing GSEA of RNA-sequencing data of the patients from TCGA database that are separated into NURR1-High and Low groups based on their ranked expression level of NURR1. The result reveal that the Wnt/β-catenin signaling is significantly enriched in NURR1-High group compared to the NURR1-Low group. **D** Expression analysis of a prostate adenocarcinoma dataset (PRAD) from TCGA database reveals that *NR4A2* exhibits a positive expression correlation with *CTNNB1* in primary prostate cancer tissues (https://www.cancer.gov/tcga). **E**, **F** RT-qPCR analysis of *CTNNB1* (β-catenin) expression in DU 145-NURR1 infectants and DU 145-shNURR1 infectants. Results showed that CTNNB1 exhibited a significant upregulation in DU 145-NURR1 infectants and conversely downregulation in DU 145-shNURR1 infectants. *P < 0.05 versus vector or scramble controls.

To further identify the cellular processes and signaling pathways as mediated by NURR1 transactivation in prostate cancer cells, we established the NURR1-overexpressed prostate cancer cell infectants derived from the DU 145 cell line (Supplementary Fig. S1A) and performed RNA sequencing. GSEA of RNA sequencing data revealed that Wnt/β-catenin signaling was significantly enriched in NURR1-overexpression (NURR1) group as compared to the control (Vector) group, indicating that Wnt/β-catenin signaling would be regulated by NURR1 (Fig. 2A, B). To further confirm these findings and reveal clinical significance, we also performed GSEA on the RNA-seq data of prostate cancer patients obtained from the TCGA database with the patients separated into NURR1-High and Low groups based on their ranked expression levels of NURR1. GSEA of RNA-sequencing data of these two patient groups further showed that Wnt/β-catenin signaling was significantly enriched in the NURR1-High group as compared to the Low group (Fig. 2B, C). Further expression analysis revealed that a positive expression correlation between NR4A2 (NURR1) and CTNNB1 (β-catenin) was shown in clinical prostate cancer tissues (Fig. 2D). Additionally, expression analyses showed that the mRNA level of CTNNB1 (β-catenin) was markedly increased in DU 145-NURR1 infectants, but significantly reduced in DU 145-shNURR1 infectants (Fig. 2E, F). Together, these results suggest that NURR1 could perform a regulatory role in Wnt/β-catenin signaling in prostate cancer.

### NURR1 functions to activate β-catenin signaling in prostate cancer cells via its direct transactivation of *CTNNB1*

**Fig. 3:**
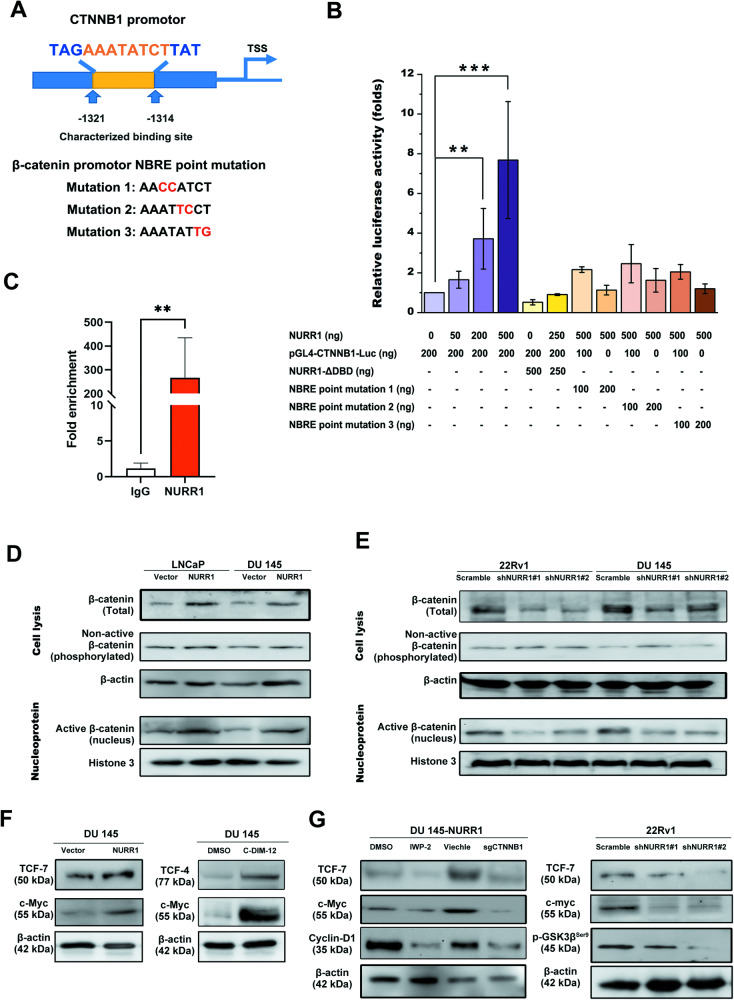
NURR1 can activate the Wnt/β-catenin signaling in prostate cancer cells via its direct transactivation of *CTNNB1* and also activation of β-catenin. **A** Schematic diagram of reporter construct driven by a 1.3 kb-fragment of *CTNNB1* promoter containing the NGFI-B response element (NBRE; AAATATCT) and also three reporter constructs containing the point-mutated NBRE. **B** Luciferase reporter assay. Results showed that only intact NURR1 but not its DBD-deleted mutant NURR1-ΔDBD could transactivate the CTNNB1 promoter-driven reporter in a dose-dependent manner. However, NURR1 could not transactivate the reporters containing the point-mutated NBRE. **C** Chromatin immunoprecipitation (ChIP) assay. Results showed that the putative NURR1-binding site on the *CTNNB1* promoter was enriched of NURR1 in HEK293 cells. IgG was set as a negative control. **D**, **E** Immunoblot analysis of β-catenin in AR-positive (LNCaP, 22Rv1) and -negative (DU 145) prostate cancer cells with either NURR1 overexpression or its knockdown. Results showed that NURR1 overexpression in LNCaP and DU 145 cells could increase, whereas its knockdown in 22Rv1 and DU 145 cells could reduce the protein levels of total β-catenin and its active form (dephosphorylated or nuclear) in target cells respectively. However, no significant changes in protein levels of non-active or phosphorylated β-catenin were seen in these prostate cancer cells with either NURR1 overexpression or its shRNA-knockdown. **F**, **G** Immunoblot analyses of downstream regulators of β-catenin signaling in DU 145 and 22Rv1 cells upon treatments with a NURR1 agonist C-DIM-12 (5 μM) or Wnt inhibitor IWP-2 (10 μM). Results showed that DU 145-NURR1 infectants or treatment of parental DU 145 cells with C-DIM-12 could increase the expressions of TCF-4, TCF-7 and c-Myc in target cells. Conversely, knockdown (shNURR1) or knockout (sgNURR1) of NURR1 in target DU 145 or 22Rv1 cells and also treatment of parental DU 145 cells with IWP-2 could reduce the expressions of TCF-7, c-Myc and cyclin D1 in these target cells.

We next sought to determine whether *CTNNB1* would be a direct target of NURR1 in target cells and NURR1 could directly transactivate the *CTNNB1* in vitro. We predicted a putative NURR1-binding site located in the *CTNNB1* promoter (Fig. 3A). Results of luciferase reporter gene assay performed in HEK293 cells showed that intact NURR1 but not its DBD-truncated mutant could significantly transactivate the CTNNB1 promoter-driven reporter in a dose-dependent manner. However, NURR1 could not transactivate reporters containing the point-mutated NURR1-binding motifs/NBRE (Fig. 3A, B). Results of ChIP assay validate that the putative NURR1-binding site located at *CTNNB1* promoter was enriched of NURR1 (Fig. 3C). Together, these results indicate that NURR1 can directly transactivate *CTNNB1* gene.

We next sought to determine whether the Wnt/β-catenin signaling transduction pathway could be activated in prostate cancer cells in a canonical manner (with increased cytoplasmic accumulation of β-catenin and nucleus translocation of active or non-phosphorylated β-catenin) in the absence of Wnt signals by immunoblot analyses. Results showed that overexpression of NURR1 could significantly increase both the total protein levels of β-catenin and also its nuclear levels of its activated form, whereas its knockdown could substantially reduce its total and nuclear levels in prostate cancer cells independent of their AR expression status (Fig. 3D, E). These results suggest that NURR1 not only could directly transactivate the *CTNNB1* and increase the protein expression levels of β-catenin but also enhance the nuclear translocation of active β-catenin to mediate the activation of β-catenin signaling in prostate cancer cells. Further immunoblot analyses also showed that overexpression of NURR1 or treatment with a NURR1 agonist C-DIM-12 [53, 54] could increase the protein expression levels of TCF-4 and TCF-7 (key transcription factors of coactivator β-catenin) and c-Myc (target of β-catenin/TCF) in DU 145 cells (Fig. 3F). Conversely, treatment with a Wnt production inhibitor IWP-2, knockout of *CTNNB1* or knockdown of NURR1 could significantly suppress the protein expression levels of TCF-7, β-catenin targets (c-Myc and cyclin D1) and phosphorylated GSK-3β^Ser9^ in DU 145 and 22Rv1 cells (Fig. 3G). Together, these results suggest that besides transactivation of *CTNNB1*, NURR1 could also activate the β-catenin-mediated signaling in prostate cancer cells via its activation (dephosphorylation) and enhanced nuclear accumulation; and through this activation, it could enhance/promote expression of its interacting transcription factors TCF and activate downstream signaling/targets with no significant change of Wnt signals.

### NURR1 can function to promote in vitro stemness features of prostate cancer cells

**Fig. 4:**
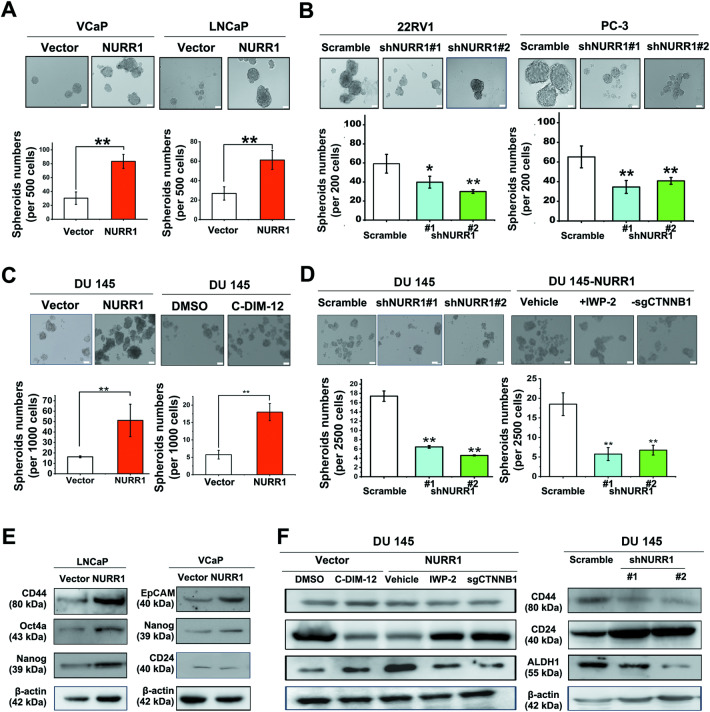
NURR1 can function to enhance in vitro stemness of prostate cancer cells. **A**–**D** Non-adherent 3D-culture spheroid formation assay. Top: Representative images of spheroids formed by various prostate cancer cell infectants. Bottom: Quantitative analysis of spheroids formed. Results showed that VCaP/LNCaP/DU 145-NURR1 infectants formed more and larger spheroids, whereas 22Rv1/PC-3/DU 145-shNURR1 infectants with NURR1-knockdown and DU 145-sgCTNNB1 infectants with CTNNB1-knockout formed less and smaller spheroids under non-adherent 3D-culture condition. *P < 0.05; **P < 0.001 versus vector or scramble-shRNA. **E**, **F** Immunoblot analyses of PCSCs-associated markers in NURR1- and shNURR1 infectants and their infectants treated with C-DIM-12 or IWP-2. Results showed that LNCaP/VCaP-NURR1 infectants and DU 145-vector infectants treated with C-DIM-12 expressed higher levels of PCSC-associated markers (including CD44, Oct4a, Nanog and EpCAM) and lower levels of CD24 (a negative marker of PCSCs). Conversely, DU 145-shNURR1 or DU 145-NURR1-sgCTNNB1 infectants and also IWP-2-treated DU 145-NURR1 infectants expressed lower levels of CD44 and ALDH1 and higher levels of CD24.

To explore the functional significance of NURR1 in the regulation of stemness in prostate cancer cells, we evaluated the anchorage-independent growth capacities of prostate cancer cells as mediated by either genetic (NURR1 overexpression or its reduction by gene knockdown or knockout) or signal activity modifications (enhancement of NURR1 activity by C-DIM-12 or suppression of Wnt production by IWP-2) by agar-based non-adherent 3D-culture assay. Results showed that NURR1 overexpression or its activation by C-DIM-12 could significantly enhance spheroid formation capacities in prostate cancer cells (VCaP, LNCaP and DU 145), whereas its knockdown/knockout or suppression of Wnt signal by IWP-2 could suppress the spheroid formation capacities in prostate cancer cells (Fig. 4A–D). Immunoblot analyses validated that NURR1 overexpression or its transactivation stimulation by C-DIM-12 could induce increased expressions of multiple PCSC-associated markers (including CD44, EpCAM, Oct4a, Nanog except CD24 which is a negative PCSC marker), whereas its reduced expression or IWP-2-induced Wnt signal suppression could reduce these PCSC-associated markers in prostate cancer cells (Fig. 4E, F). RT-qPCR analysis also showed that NURR1-overexpression or its knockdown could elevate or decrease the PCSC-associated and EMT markers in different infectants (Supplementary Fig. S2). Together, these results suggest that NURR1 could function to promote the in vitro stemness potential of prostate cancer cells.

### NURR1 can function to promote castration-resistance of prostate cancer cells in vivo

**Fig. 5:**
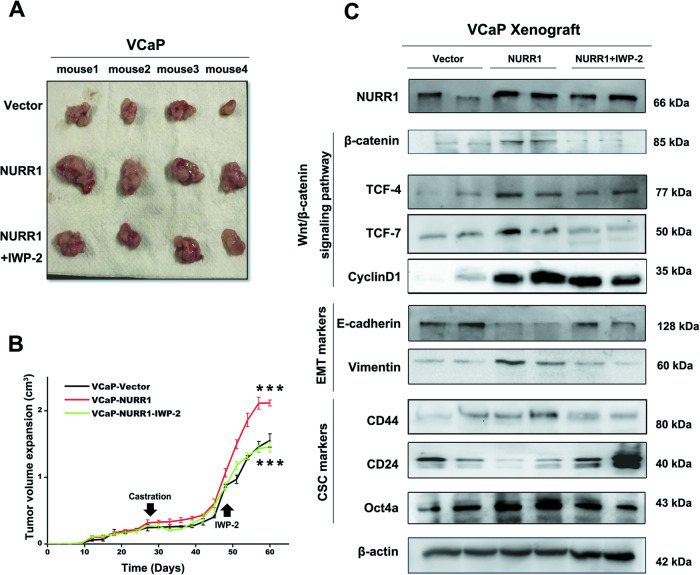
NURR1 can function to enhance castration-resistant growth of prostate cancer cells in vivo. **A**, **B** In vivo tumorigenicity assay. **A** Images of dissected xenograft tumors grown in host mice (5-week post-castration) by VCaP-NURR1 and VCaP-vector infectants, and also VCaP-NURR1 infectants with IWP-2 treatment. **B** Semi-quantitative analysis of sizes of xenograft tumors grown in castrated mice by NURR1 Infectants with or without IWP-2 treatment. Results showed that in 28-day post-castration growth relapse, VCaP-NURR1 infectants grew tumors at faster rate than VCaP-vector infectants in castrated mice. However, treatment with IWP-2 could significantly suppress the castration-relapse tumor growth of VCaP-NURR1 infectants in castrated mice. **C** Immunoblot analyses of castration-relapse xenograft tumors formed by VCaP-NURR1 infectants. Results showed that castration-rebound VCaP-NURR1 tumors expressed higher levels of β-catenin and also enhanced levels of phenotypic markers of EMT (reduced E-cadherin and increased vimentin levels) and cancer stemness (increased CD44 and Oct4a, and decreased CD24 levels). However, expression levels of these phenotypic markers were either reduced or reversed upon treatment of host mice with IWP-2. **P < 0.01 VCaP-NURR1 versus VCaP-vector.

We next determined the significance of NURR1 overexpression in castration-relapse growth of prostate cancer cells using an established xenograft model of CRPC, that is based on the castration-relapse growth of AR-positive VCaP cells [34]. Results of in vivo tumorigenicity assay demonstrated that in 28-day post castration, xenograft tumors formed by VCaP-NURR1 infectants continued to grow at faster rates than that of control vector infectants (Fig. 5A, B). Notably, castration-relapse growth of VCaP-NURR1 xenografts could be moderately repressed by in vivo treatment with Wnt inhibitor IWP-2. To provide further insight into the malignant growth features of xenograft tumors, we examined the expression status of markers of Wnt/β-catenin signaling, EMT and cancer stemness in castration-rebound tumors formed by VCaP-NURR1 infectants in castrated mice by immunoblot analyses. Results confirmed that castration-relapse VCaP-NURR1 xenografts expressed significant higher protein level of β-catenin than that in VCaP-vector xenografts. Expression results also showed that castration-rebound VCaP-NURR1 tumors expressed enhanced levels of phenotypic markers of EMT (reduced E-cadherin and increased vimentin levels) and cancer stemness (increased CD44 and Oct4a, and decreased CD24 levels) (Fig. 5C). On the other hand, in vivo treatment of castrated host mice with IWP-2 could significantly suppress or abolish β-catenin expression in castration-relapse tumors. IWP-2 treatment could also reduce the expressions of transcription factors TCF-4 and TCF-7 and β-catenin/TCF target cyclin D1 in castration-relapse VCaP-NURR1 tumors, suggesting that the enhanced Wnt/β-catenin signaling in VCaP-NURR1 tumors was suppressed by the treatment. Finally, IWP-2 treatment could also attenuate the expressions of markers of EMT (enhanced E-cadherin and reduced vimentin levels) and cancer stemness (reduced CD44 and Oct4a but increased CD24 levels) as seen in the castration-relapse VCaP-NURR1 tumors. Together, these results indicate that NURR1 could function to enhance the castration-resistant growth of prostate cancer cells in vivo, mediated through its activation of β-catenin activity accompanied with enhanced EMT and cancer stemness; and also targeting Wnt signaling could suppress the NURR1-induced castration-relapse tumor growth of prostate cancer.

### NURR1 could function to promote migration, invasion and metastasis potential of prostate cancer cells

**Fig. 6:**
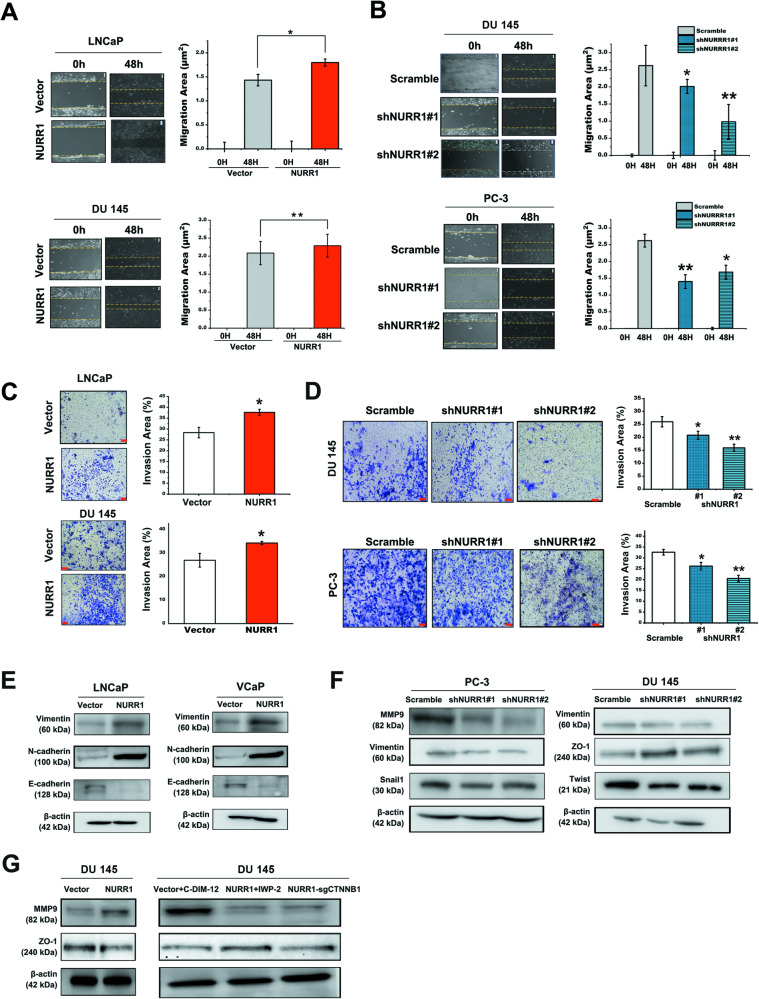
NURR1 can promote in vitro migration and invasion potential of prostate cancer cells. **A,** **B** Wound healing assay. Left: Representative images of scratched and recovered wound areas (demarcated by dotted lines) on confluence monolayers of infectants of NURR1, shNURR1 and their controls taken at 0- and 48-h time points. Right: Semi-quantitative analysis of wound closure. Results showed that LNCaP-NURR1 and DU 145-NURR1 infectants exhibited significant higher migration potential, whereas DU 145-shNURR1 and PC-3-shNURR1 infectants displayed slower migration potential as compared to their corresponding controls. **C**, **D** Transwell-invasion assay. Left: Representative images of invaded cells. Right: Quantification analysis of field area occupied by invaded cells (% of total field area). Results showed that LNCaP-NURR1 and DU 145-NURR1 infectants manifested significant higher invasion potential, whereas DU 145-shNURR1 and PC-3-shNURR1 infectants showed lower invasion potential as compared to their control infectants. **E**, **F** Immunoblot analysis of EMT markers in NURR1- and shNURR1 infectants. Results showed that LNCaP-NURR1 and VCaP-NURR1 infectants expressed enhanced levels of EMT markers (increased vimentin and N-cadherin levels, suppressed E-cadherin level), whereas PC-3-shNURR1 and DU 145-shNURR1 infectants displayed the reversed profile of these markers (reduced vimentin, snail1, twist and MMP9 levels; increased ZO-1 level). **G** Immunoblot analysis of EMT markers in DU 145-NURR1 infectants treated with C-DIM-12/IWP-2 or plus CTNNB1 knockout. Results showed that treatment with C-DIM-12 or IWP-2 induced significant loss of epithelial cell marker ZO-1 expression and comparable high level of MMP9 as in untreated DU 145-NURR1 infectants. Knockout of CTNNB1 could attenuate the expression levels of MMP9 and ZO-1 in DU 145-NURR1 infectants. *P < 0.05; **P < 0.01 versus vector or scramble controls.

Results of GSEA as revealed from the prostate cancer patients derived from TCGA database (Fig. 2C) and the expression patterns of representative EMT biomarkers in NURR1-overexpression prostate cancer cells (Fig. 5C) suggest that NURR1 could play a supportive role in EMT regulation in prostate cancer cells. We next examined the functional significance of NURR1 in EMT-regulated migration and invasion capacities of prostate cancer cells. In vitro phenotypic characterization analyses showed that overexpression of NURR1 could enhance, whereas its knockdown could suppress both the migration and invasion capacities of prostate cancer cells (Fig. 6A–D). Immunoblot analyses validated that overexpression of NURR1 could enhance the expressions of multiple mesenchymal cell markers (including N-cadherin, vimentin, MMP9) but suppress the expressions of epithelial cell markers (E-cadherin, ZO-1) in prostate cancer cells; conversely, its knockdown could reverse the expressions of these EMT markers in prostate cancer cells (Figs. 6E, F). We also examined the effects of either NURR1 activation or suppression of Wnt signaling on the expressions of EMT markers in DU 145 cells. Immunoblot results showed that treatment of DU 145-vector control cells with NURR1 agonist C-DIM-12 could significantly suppress or abolish the expression of ZO-1 as seen in DU 145-NURR1 infectants. Similarly, treatment with Wnt inhibitor IWP-2 or knockout of *CTNNB1* in DU 145-NURR1 infectants could also induce a similar reduction of ZO-1 expression as in DU 145-NURR1 infectants (Fig. 6G).

**Fig. 7:**
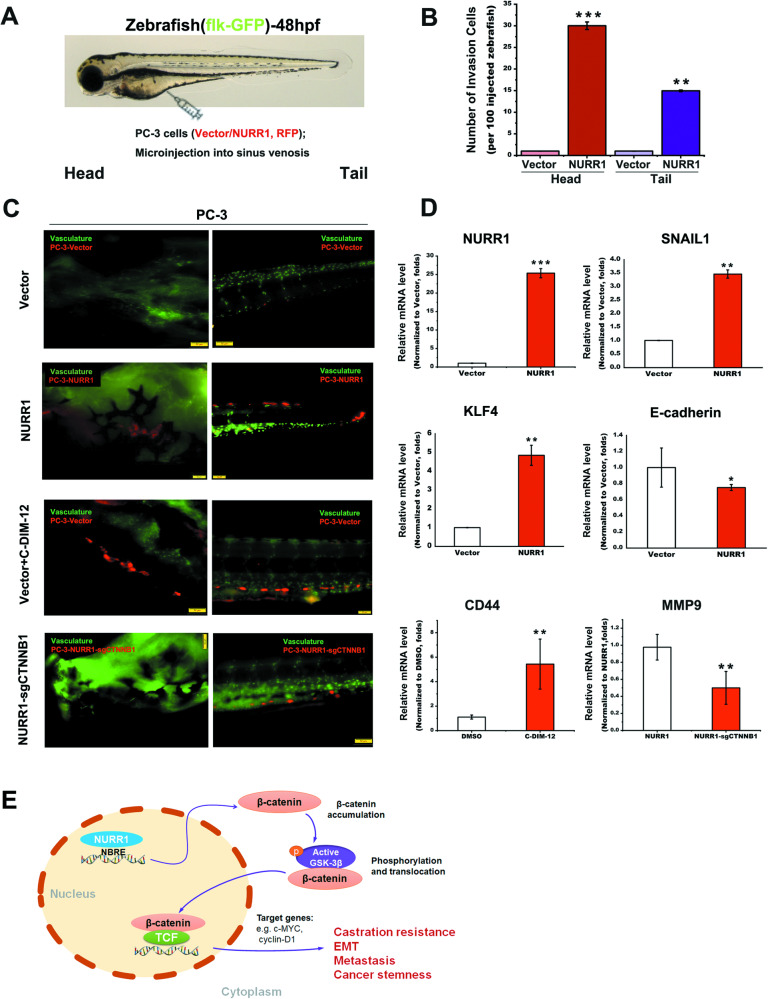
NURR1 can function to enhance in vivo metastasis potential of prostate cancer cells as assayed using a zebrafish embryo model. **A** Picture illustrates the microinjection of RFP-labeled PC-3-NURR1/vector infectants into the developing heart of flk:GFP zebrafish fry (48-h post fertilization). **B** Fluorescent microscopic images of migrated PC-3-NURR1/vector infectants detected in cranial (head) and caudal (tail) regions of zebrafish at 4-day post-injections. Green: blood vessels; red: PC-3 infectants. Microscopic examination showed that at 4-day post injections, significant number of PC-3-NURR1 infectants were detected in both head and tail regions of zebrafish, whereas almost absence of PC-3-vector infectants were detected in these regions. However, significant number of PC-3-vector infectants were detected in zebrafish upon treatment with C-DIM-12. On the other hand, no PC-3-NURR1 infectants with *CTNNB1* knockout were detected in cranial and caudal regions of zebrafish but with few cells detected in the blood vessels. **C** Quantification analysis of PC-3-NURR1/vector infectants migrated to head and tail regions of zebrafish. **D** RT-qPCR analysis of EMT markers expressed in PC-3-NURR1/vector infectants as migrated to distal sites of zebrafish. Results showed that the migrated PC-3-NURR1 infectants exhibited enhanced expression profiles of EMT markers (increased SNAIL1 and KLF4 levels and decreased E-cadherin level) as compared to that in PC-3-vector infectants. Treatment of zebrafish with C-DIM-12 could significantly increase CD44 expression. Migrated PC-3-NURR1 infectants with CTNNB1 knockout showed reduced MMP9 expression. *P < 0.05; **P < 0.01; ***P < 0.001 versus vector or scramble controls. **E** Schematic diagram depicts the characterized role of NURR1 in the promotion of castration-resistance, metastasis and cancer stemness in advanced prostate cancer via its direct targeting of *CTNNB1* (β-catenin).

To evaluate the significance of NURR1 in metastasis potential of prostate cancer cells, we next performed the in vivo metastasis assay on injected PC-3-NURR1/vector infectants in a zebrafish embryo model. Results showed that the injected PC-3-NURR1 infectants exhibited significant higher metastasis potential to the developing brain and caudal regions of zebrafish as compared to control PC-3-vector infectant (Fig. 7A, B). In vivo C-DIM-12 treatment of zebrafish bearing the injected PC-3-vector infectants could also enhance the metastasis potential of injected cells migrated to the distal sites (Fig. 7C). Conversely, knockout of *CTNNB1* in PC-3-NURR1 cells could significantly suppress or abolish the metastasis potential of injected cells in zebrafish (Fig. 7C). We also analyzed the expression status of representative EMT markers in the migratory PC-3-NURR1/vector infectants in the cranial and caudal parts of zebrafish by RT-qPCR assay. Results showed that a consistent increased expression pattern of mesenchymal cell markers (SNAIL1 and KLF4) and a decreased expression of epithelial cell marker E-cadherin were detected in the migratory PC-3-NURR1 infectants as observed in the in vitro assays (Fig. 7D). Similarly, RT-qPCR analysis on the dissected cranial and caudal parts of zebrafish containing the migratory infectants also demonstrated that in vivo C-DIM-12 treatment of zebrafish embryo with injected PC-3-vector infectants showed an increased expression of an EMT/CSC-associated marker CD44 whereas knockout of *CTNNB1* in PC-3-NURR1 infectants showed a decreased expression of mesenchymal cell marker MMP9 (Fig. 7D). These results suggest that NURR1 could function to promote the in vivo migration or metastasis potential of prostate cancer cells.

## Discussion

In the present study, we demonstrated that orphan nuclear receptor NURR1 (*NR4A2*), which displays an upregulation in prostate cancer, could act to promote both in vitro (cancer stemness and EMT features) and in vivo (castration resistance and metastasis capacities) oncogenic growth of prostate cancer cells via its direct transactivation of *CTNNB1*, resulted in increased level of activated or nuclear β-catenin and subsequent activation of Wnt/β-catenin signaling pathway in prostate cancer cells regardless of their AR expression status (Fig. 7E).

Aberrant activation of Wnt/β-catenin signaling and mutations of pathway members can contribute to different stages of prostate carcinogenesis, with that it helps to promote cancer stemness, EMT and metastasis in advanced metastatic CRPC [55]. Studies indicate that interplay or dysregulation of signalings between β-catenin and androgen receptor (AR) can contribute to the advanced and androgen-independent growth of prostate cancer. It is shown that β-catenin can act as an AR coactivator and its recruitment to either ligand-activated or non-activated AR can augment its transcriptional activity in prostate cancer cells [56, 57]. It is also shown that *AR* gene is the direct target of β-catenin/TCF/LEF transcriptional complex in prostate cancer cells [58]. On the other hand, overexpression of AR can potentiate Wnt/β-catenin signaling in prostate cancer cells under castrate levels of androgen and activated Wnt signaling can recruit AR to the promoters of Wnt target genes [20]. An interplay between AR and Nur77 (a member of NR4A subfamily) is shown in mouse and human granulosa cells [59]. Nur77 can bind to the NGFI-B response element (NBRE) in the AR promoter and its overexpression can upregulate AR expression in granulosa cells. However, the putative interplay between AR and any member of NR4A in prostate cancer cells remains to be demonstrated. Our preliminary study observed that overexpression of NURR1 could increase protein expression of AR and activation of AR signaling in prostate cancer cells (unpublished results), suggesting that there could be a direct positive regulation between two nuclear receptors in prostate cancer cells and with this interaction contributing to potentiation of AR signaling in advanced growth of prostate cancer. However, the mechanism involved in this crosstalk remains to be further validated.

Interplay between NURR1 and β-catenin signaling has been shown in non-prostatic target cells. An in vitro study/analysis in human osteosarcoma U2-OS cells shows that NURR1 can transcriptionally repress β-catenin-mediated transactivation of Axin2, a degradation or negative β-catenin signaling regulator, and inversely β-catenin can repress the transcriptional activity of NURR1, suggesting a negative reciprocal regulatory loop is present between the two factors [60]. Another in vitro study in rat pheochromocytoma PC12 cells shows that NURR1 protein can physically interact with β-catenin and β-catenin can bind to the NURR1 promoter; and with co-activation of β-catenin and NURR1 is implicated in the functional maintenance of midbrain dopaminergic neurons [61]. A functional crosstalk between another member of NR4A subfamily NR4A1 (Nur77, NGFI-B) and β-catenin in EMT mediation has been shown in other cancers. Wu et al. show that β-catenin can act to induce Nur77 expression in colon cancer cells by transactivation of Nur77 promoter via its activation of AP-1 transcription factor [62]. In breast cancer cells, TGF-β-induced p38 kinase activity can phosphorylate NR4A1 and TGF-β-induced β-catenin can transactivate NR4A1 expression via formation of β-catenin/TCF/LEF complex and its binding to NR4A1 promoter [63]. It remains to further elucidate whether a reciprocal regulatory loop between NURR1 and β-catenin could be present in prostatic cells.

Androgen-deprivation therapy or AR-axis targeted therapy, aiming to suppress AR or androgen signaling, is still the major therapeutic option for advanced prostate cancer. However, targeting AR or androgen biosynthesis still inevitably results in drug resistance and disease recurrence. Therefore, targeting oncogenic pathways other than AR signaling or their combined targeting could in principle be an effective therapeutic approach for treating metastatic or therapy-resistant prostate cancer. Here, our results showed that inhibition of Wnt production or signal by IWP-2 could suppress the in vitro growth of PCSCs and in vivo castration-relapse growth of VCaP-NURR1 xenografts, suggesting that inhibition of Wnt pathway could be of therapeutic value for treatment of CRPC. Previous studies show that inhibition of β-catenin activity by small molecules can inhibit the growth of prostate cancer cells and their resistant growth to antiandrogen [64, 65]. Our results also showed that activation of NURR1 activity by an agonist C-DIM-12 could potentiate in vitro growth of PCSCs and in vivo metastasis potential of prostate cancer cells. However, it still remains to demonstrate whether suppression of NURR1 activity by inverse agonists or inhibitors could be of any potential therapeutic application for advanced prostate cancer management.

## Conclusions

In summary, our present study demonstrates a direct role of nuclear receptor NURR1 in the promotion of both EMT and cancer stemness in prostate cancer through its direct transcriptional control of *CTNNB1* and also activation of β-catenin signaling. Moreover, our results also implicated that targeting of NURR1 or its mediation of β-catenin signaling could have a potential therapeutic application in the management of CRPC.

### Supplementary information

Authorship Change Approvals combined

Supplementary Figures S1-S2 and Tables 1-2

Full and uncropped western blots

## Data Availability

All data responsible for evaluating the conclusions in this study are presented in the paper and/or the Supplementary Materials. The datasets used and analyzed in the study are available from the corresponding author upon reasonable request.
