# Supplementary material for: Nuclear receptor NURR1 functions to promote stemness and epithelial-mesenchymal transition in prostate cancer via its targeting of Wnt/β-catenin signaling pathway
Source: Cell Death Dis. 2024 Mar 26;15(3):234. doi: 10.1038/s41419-024-06621-w (PMC10965960; doi:10.1038/s41419-024-06621-w)
Supplement: Supplementary file 1 — Authorship Change Approvals combined [file 41419_2024_6621_MOESM1_ESM.pdf]

Nuclear receptor NURR1 functions to promote stemness and epithelial-mesenchymal transition in prostate cancer via its targeting of Wnt/β-catenin signaling pathway

Xingxing Zhang[1,#], Haolong Li[1,#], Yuliang Wang[1,#], Hui Zhao[1], Zhu Wang[2,*] and Franky Leung Chan[1,*]

Authors' affiliations: [1]School of Biomedical Sciences, Faculty of Medicine, The Chinese University of Hong Kong, Hong Kong, China; [2]Department of Urology, People's Hospital of Longhua, Southern Medical University, Shenzhen, Guangdong, China

Supplementary Figures

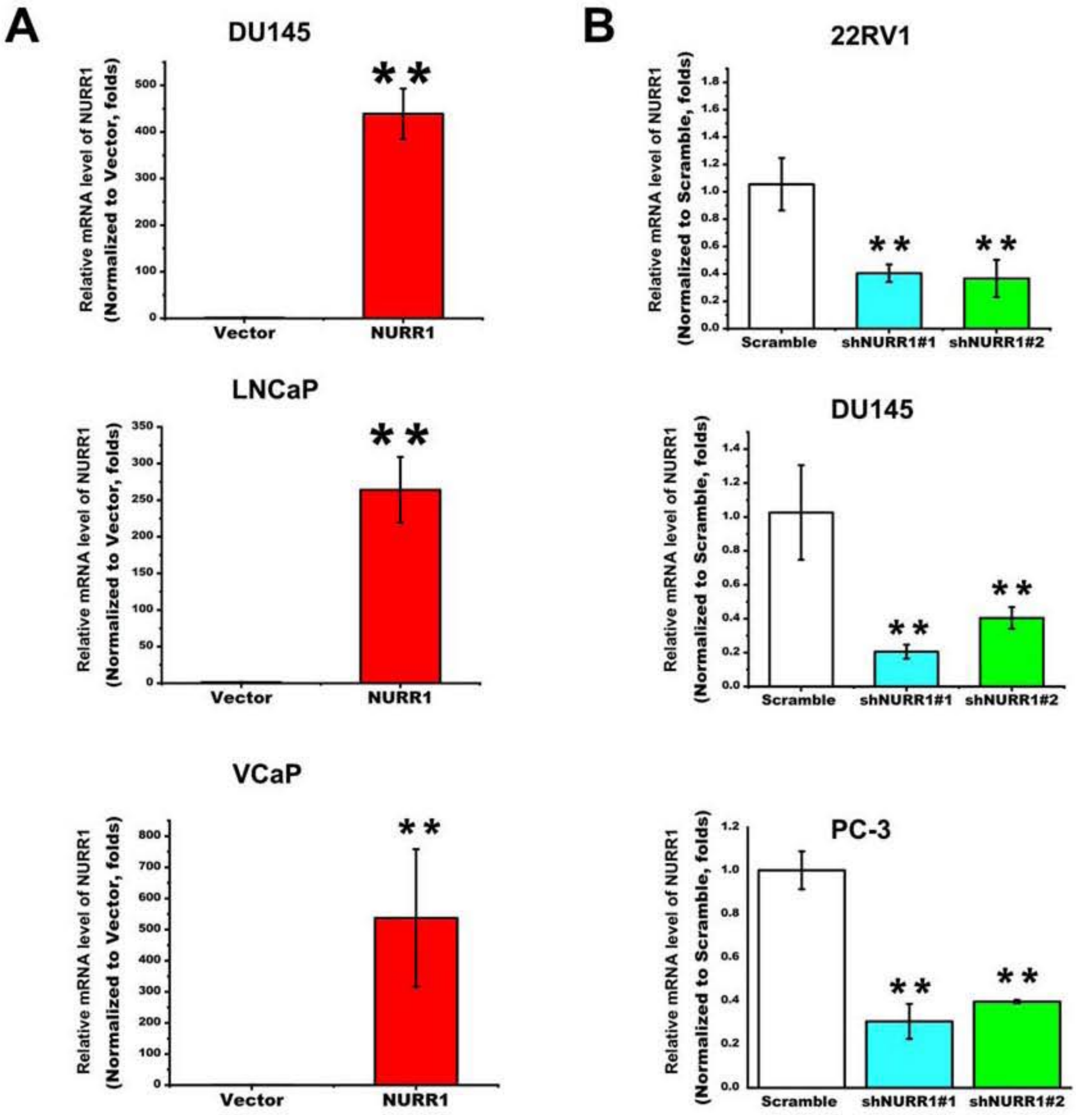

Supplementary Figure S1

Supplementary Figure S1. RT-qPCR validation of NURR1 expression in NURR1-overexpression and knockdown prostate cancer cells. (**A**) RT-qPCR detection of the NURR1 mRNA level in NURR1-overexpressed DU 145, LNCaP and VCaP cells. (**B**) RT-qPCR detection of the NURR1 mRNA level in NURR1-knockdown DU 145, 22Rv1 and PC-3 cells.

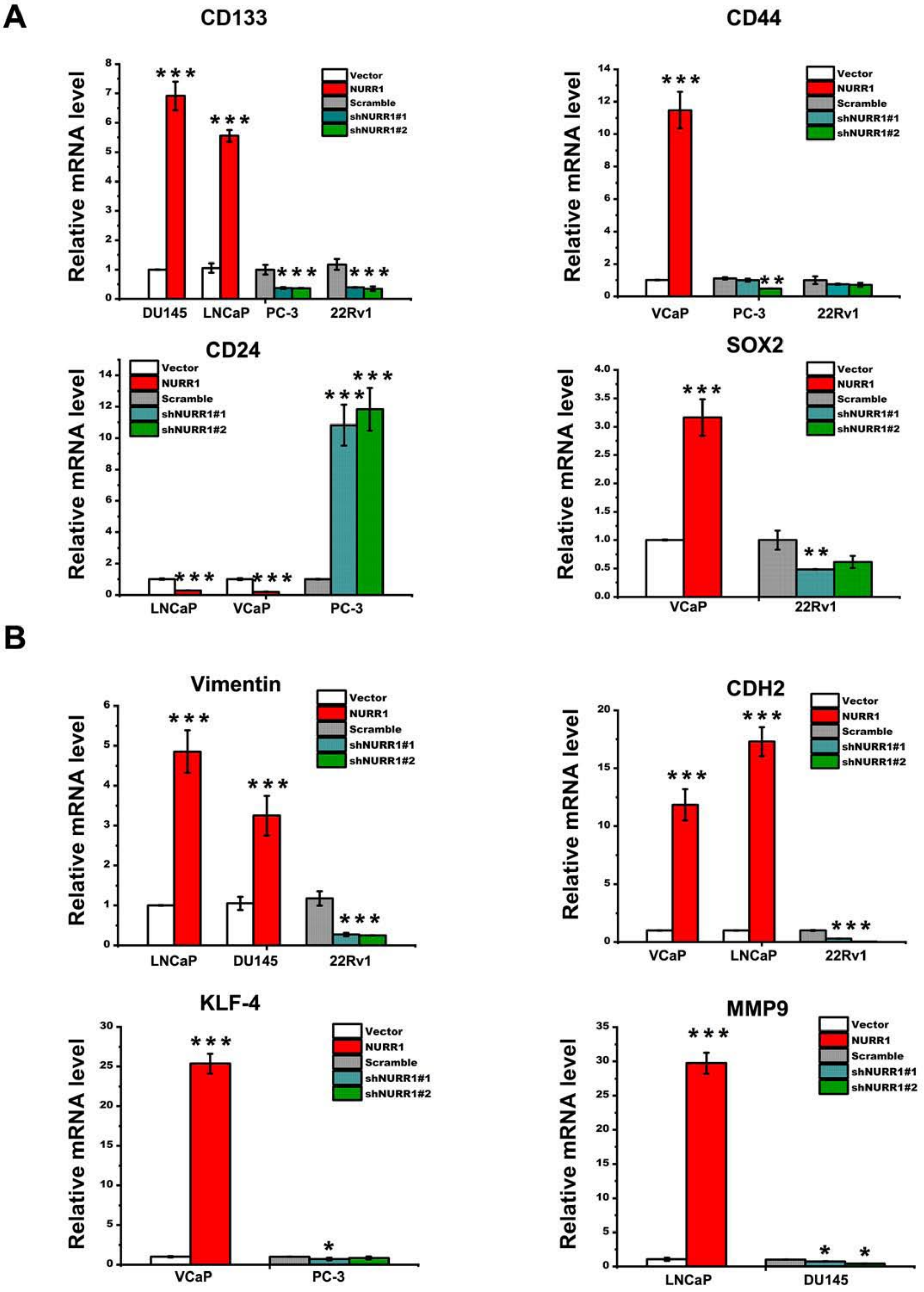

Supplementary Figure S2

Supplementary Figure S2. RT-qPCR analysis of the expression of CSC and EMT associated markers in NURR1-overexpression and -knockdown prostate cancer cells. (**A**) RT-qPCR detection of the expression of CSC markers, CD133, CD44, CD24 and SOX2 in NURR1-overexpressed DU 145, LNCaP, VCaP cells and NURR1-knockdown PC-3 and 22Rv1 cells. (**B**) RT-qPCR detection of the expression of EMT markers, Vimentin, CDH2, KLF-4 and MMP9 in NURR1-overexpressed DU 145, LNCaP, VCaP cells and NURR1-knockdown DU 145, PC-3 and 22Rv1 cells.

Supplementary Table 1

mRNA	Nucleotide sequences		GenBank accession No.
β-actin	Forward	ATGGATGATGATATCGC CGCG	NM_001101
	Reverse	CTCCATGTCGTCCCAGT TGGT	
NURR1	Forward	GGCGAACCCTGACTATCAAA	NM_006186
	Reverse	CTGGGTTGGACCTGTATGCT	
OCT3/4	Forward	GACAACAATGAAAATCT TCAGGAGA	NM_002701
	Reverse	CTGGCGCCGGTTACAGA ACCA	
NANOG	Forward	TTTGTGGGCCTGAAGAA AACT	NM_024865
	Reverse	AGGGCTGTCCTGAATAA GCAG	
SOX2	Forward	GCCGAGTGGAAACTTTT GTCG	NM_003106
	Reverse	GGCAGCGTGTACTTATC CTTCT	
CD44	Forward	GCGGCTCCTCCAGTGAAA	NM_000610

	Reverse	AGCCTGCTGAGATGGTATTT	
CD133	Forward	AAACAGTTTGCCCCCAGGAA	NM_006017
	Reverse	ACAATCCATTCCCTGTGCGT	
KLF4	Forward	TATGACCCACACTGCCAGAA	NM_001314052
	Reverse	TGGGAACTTGACCATGATTG	
E-cadherin	Forward	TCCCATCAGCTGCCCAGAAA	NM_004360.3
	Reverse	TGACTCCTGTGTTCCTGTTA	
Vimentin	Forward	ACGCCATCAACACCGAGT	NM_003380.3
	Reverse	GTGCCAGAGACGCATTGTC	
EPCAM	Forward	GCAGGGTCTAAAAGCTGGTG	NM_002354.2
	Reverse	CCCTATGCATCTCACCCATC	
SNAIL1	Forward	CGAAAGGCCTTCAACTGC AAAT	NM_005985.3
	Reverse	ACT GGTACTTCTTGACATCTG	
CD24	Forward	CTCCTACCCACGCAGATTT ATTC	NM_001291737.1
	Reverse	TGGTGGCATTAGTTGGAT TTGG	
CTNNB1	Forward	CACAAGCAGAGTGCTGAAGGTG	NM_001098209.2
	Reverse	GATTCCTGAGAGTCCAAAGACA G	

Supplementary Table 2

Target	Brand	Usage
NURR1	Abcam, ab60149	WB, IHC, ChIP
β-catenin	Cell Signaling Technology, #9562	WB
Phospho-β-catenin	Abclonal, AP0579	WB
Non-phospho (Active) β-catenin	Cell Signaling Technology, #8814	WB
TCF4	Abclonal, A12017	WB
TCF7	Abclonal, A3091	WB
ZEB1	Abclonal, A5600	WB
c-Myc	Abclonal, A1309	WB
CyclinD1	Santa Cruz Biotechnology, SC-8396	WB
Wnt3a	Abclonal, A0642	WB
Phospho-GSK3β-S9	Abclonal, AP0039	WB
Vimentin	Cell Signaling Technology, #3295	WB
N-cadherin	Cell Signaling Technology, #4061	WB
E-cadherin	Cell Signaling Technology, #4065	WB
KLF4	Cell Signaling Technology, #4038	WB
MMP-9	Cell Signaling Technology, #3852	WB
Snail	Abclonal, A5243	WB
ZO-1	Abclonal, A0659	WB
Twist	Abclonal, A7314	WB
β-actin	Santa Cruz Biotechnology, SC-47778	WB
