# Supplementary material for: Nuclear receptor NURR1 functions to promote stemness and epithelial-mesenchymal transition in prostate cancer via its targeting of Wnt/β-catenin signaling pathway
Source: Cell Death Dis. 2024 Mar 26;15(3):234. doi: 10.1038/s41419-024-06621-w (PMC10965960; doi:10.1038/s41419-024-06621-w)
Supplement: Supplementary file 2 — Supplementary Figures S1-S2 and Tables 1-2 [file 41419_2024_6621_MOESM2_ESM.pdf]

# Full and uncropped western blots

Figure 1E

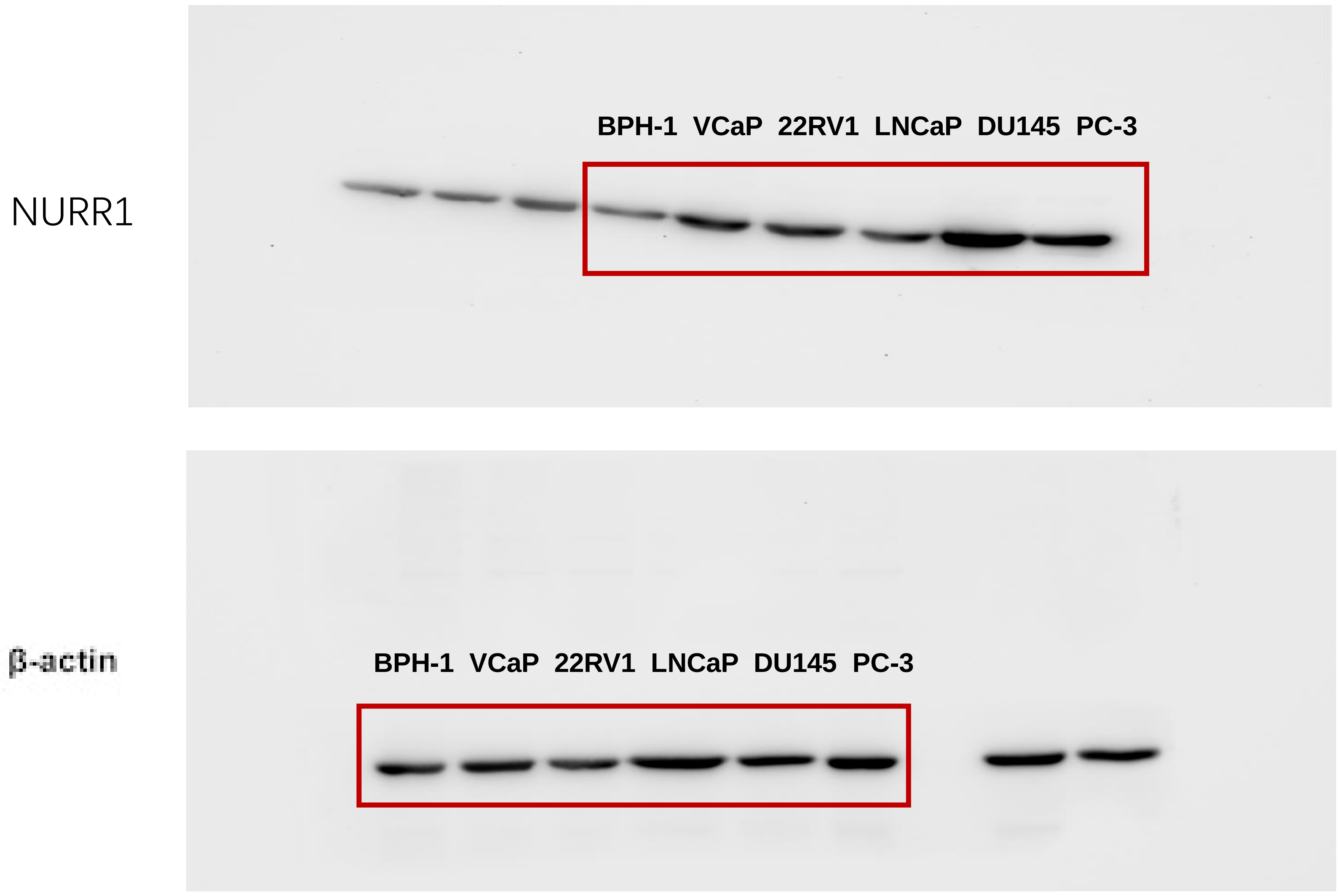
NURR1
BPH-1 VCaP 22RV1 LNCaP DU145 PC-3
β-actin
BPH-1 VCaP 22RV1 LNCaP DU145 PC-3

Figure 3D cell lysis

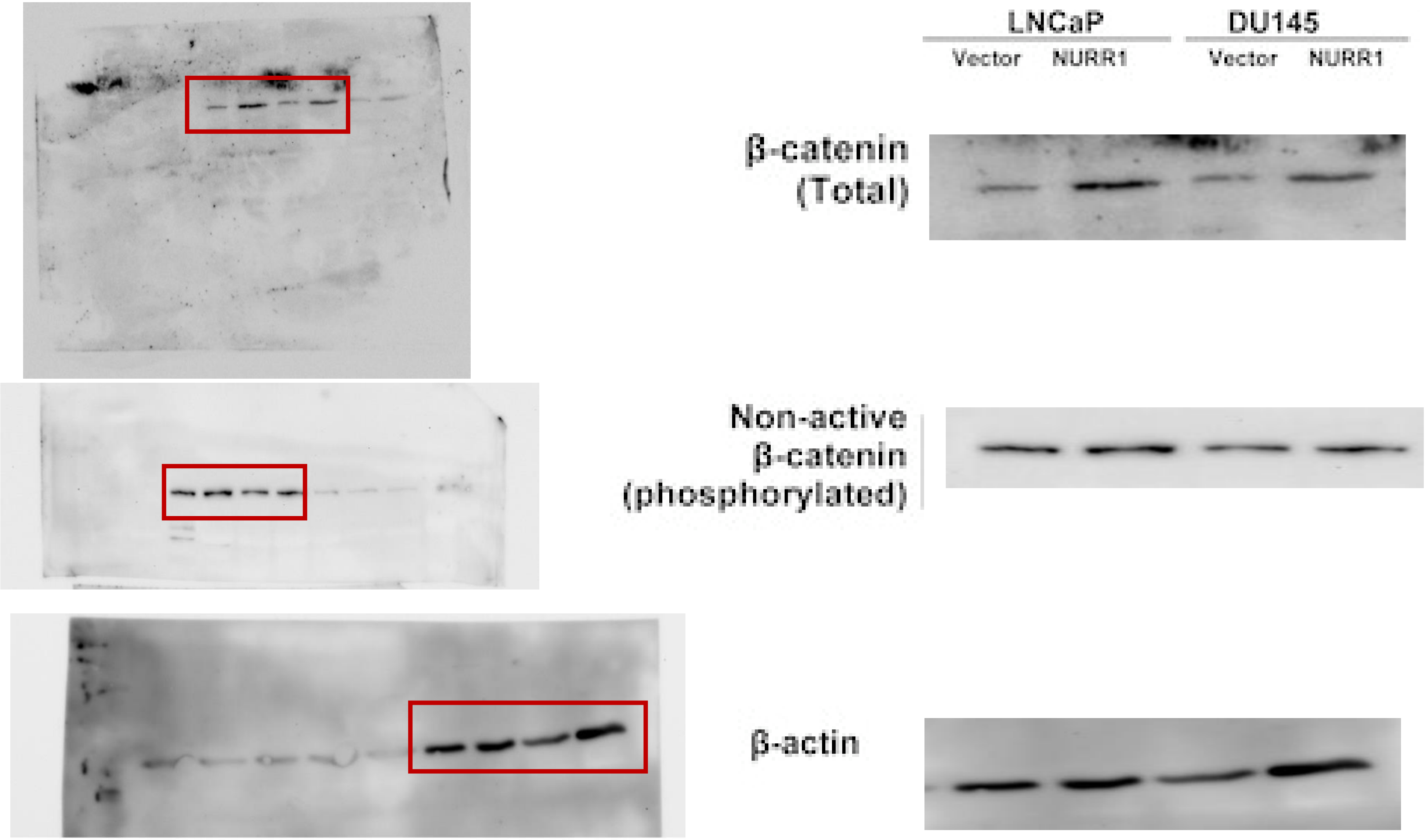

Figure 3D Nucleoprotein

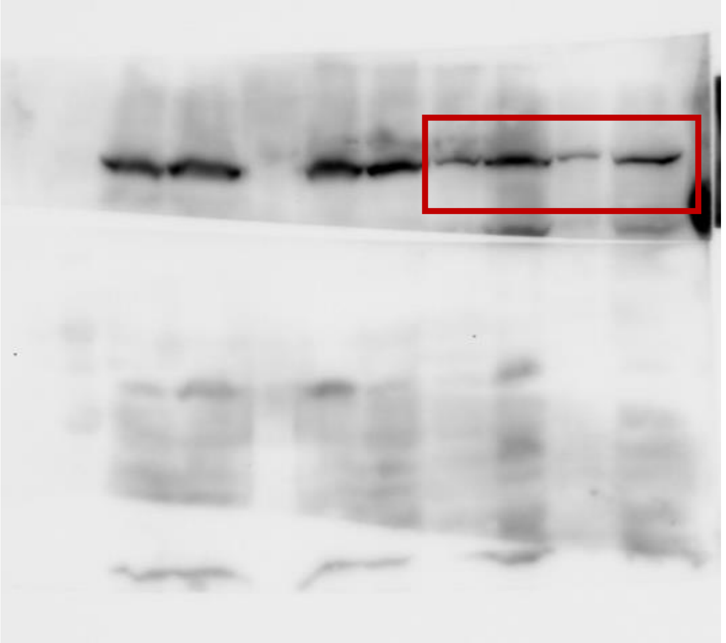

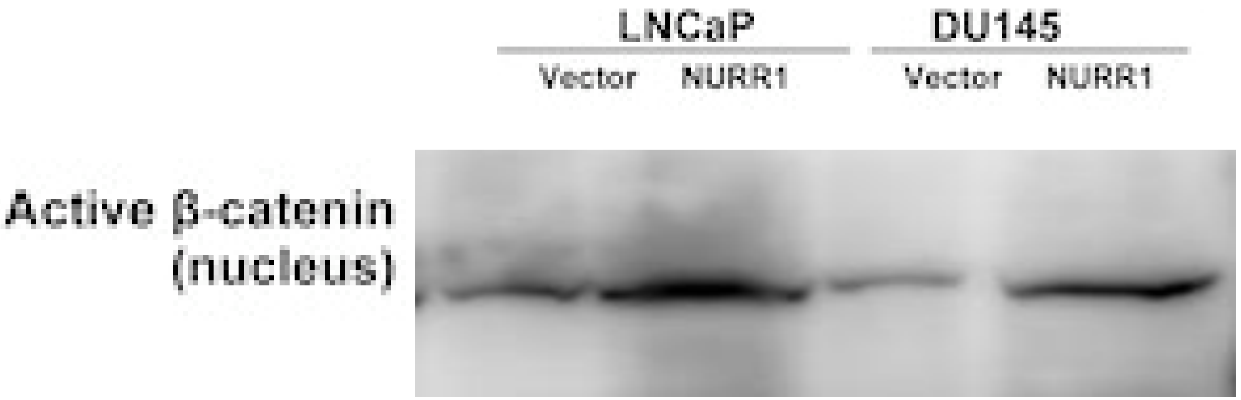


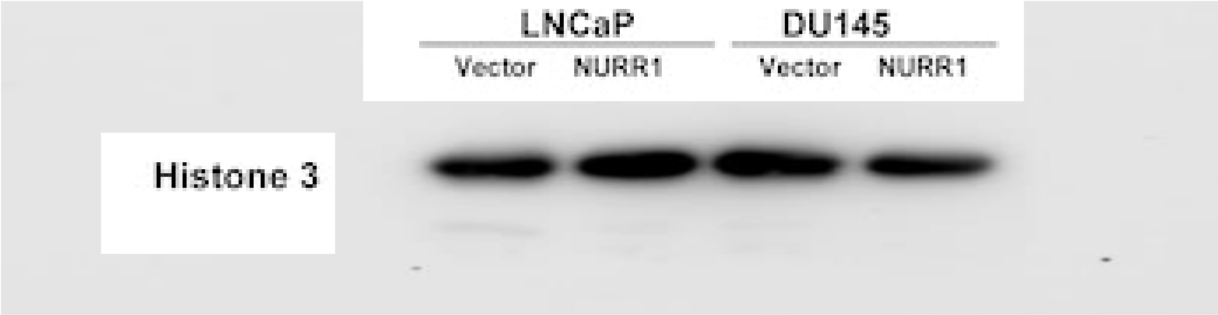

Figure 3E cell lysis

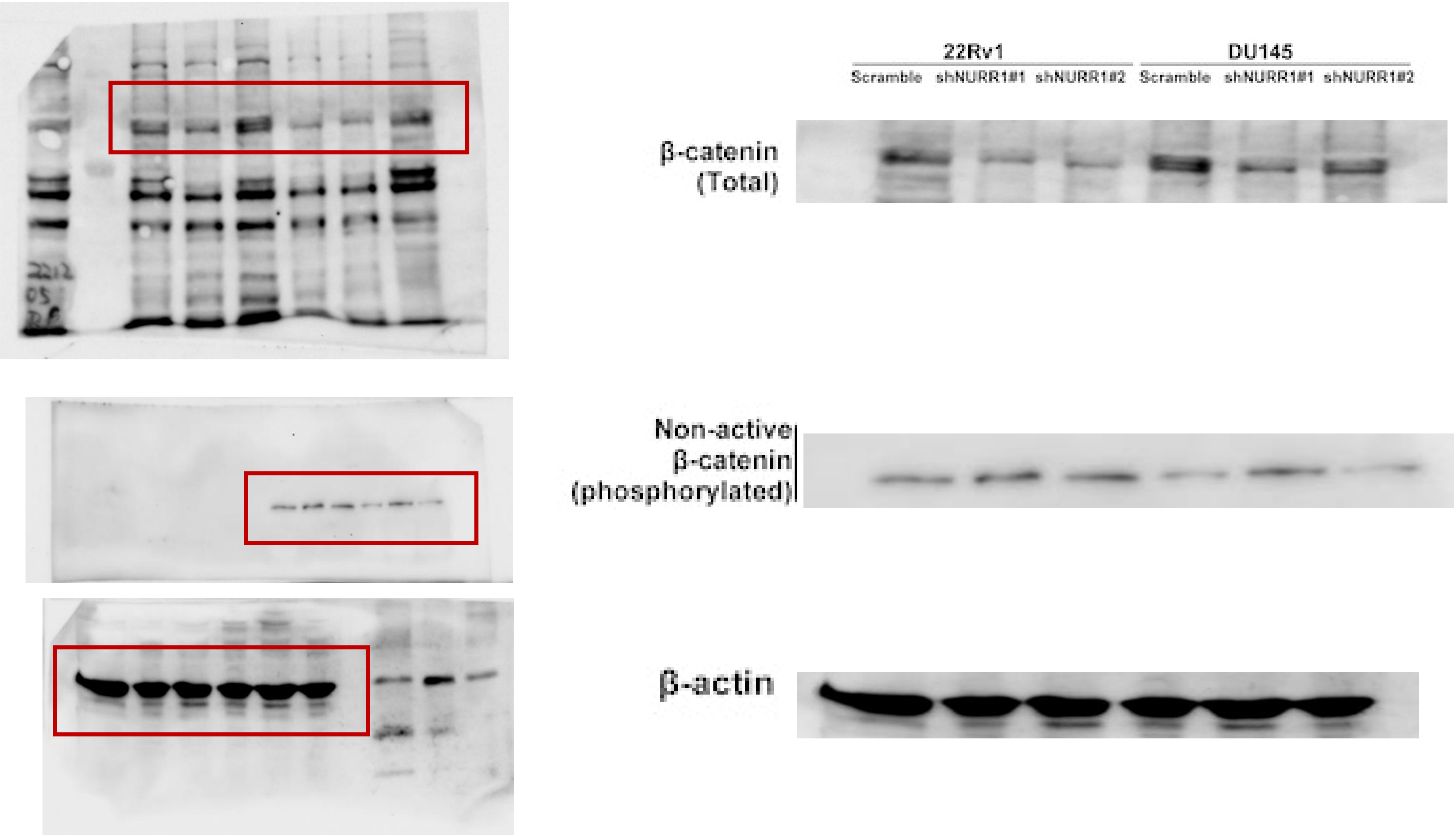

Figure 3E Nucleoprotein

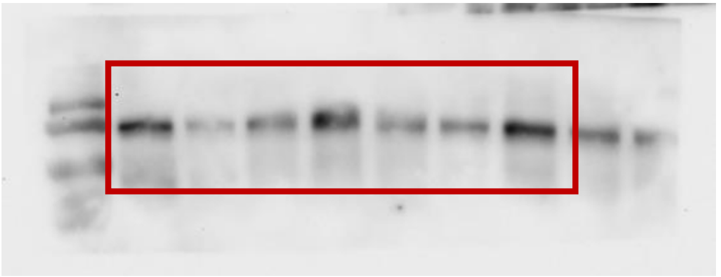

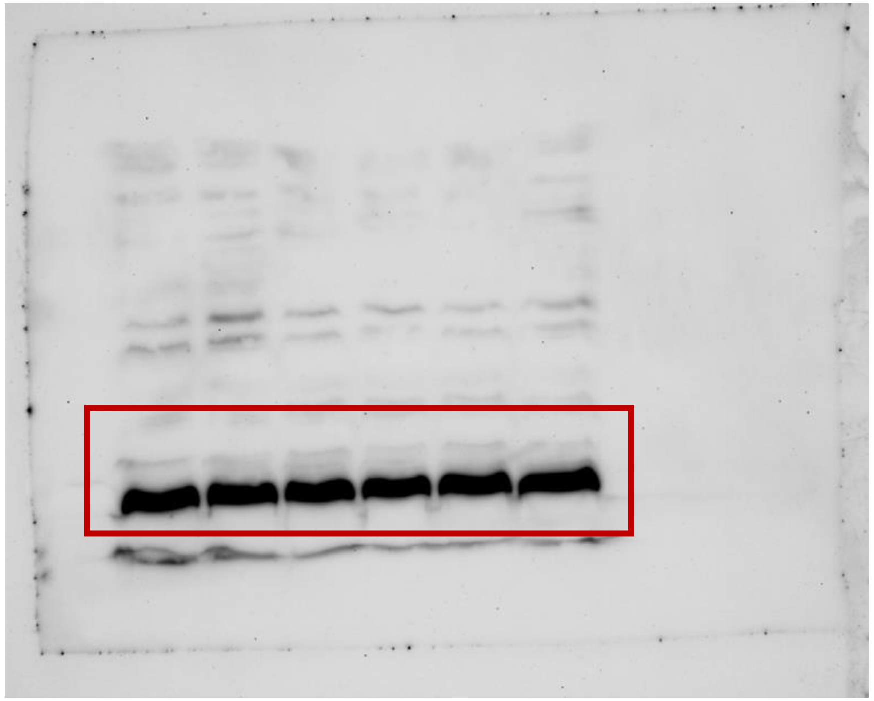

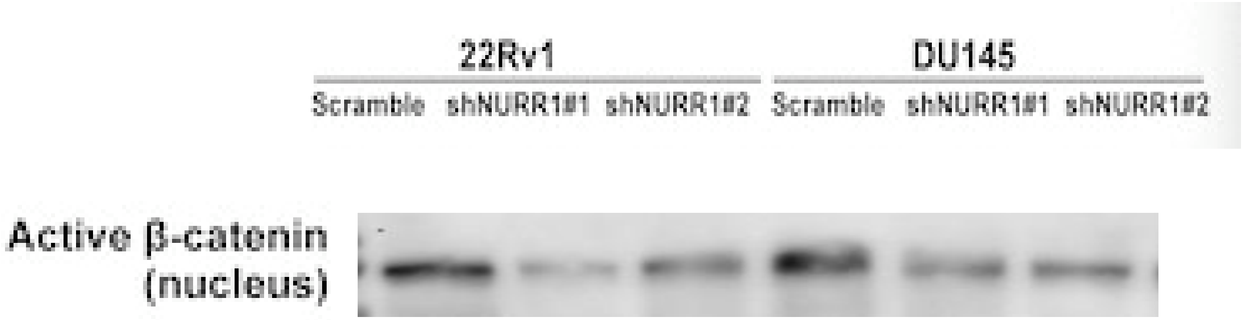


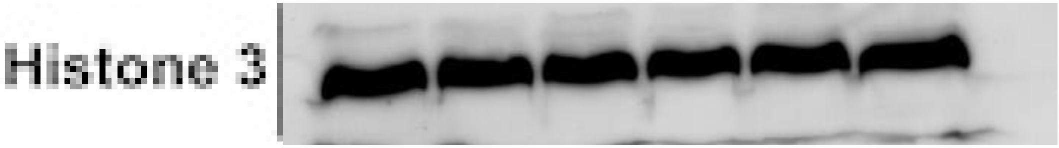

Figure 3F

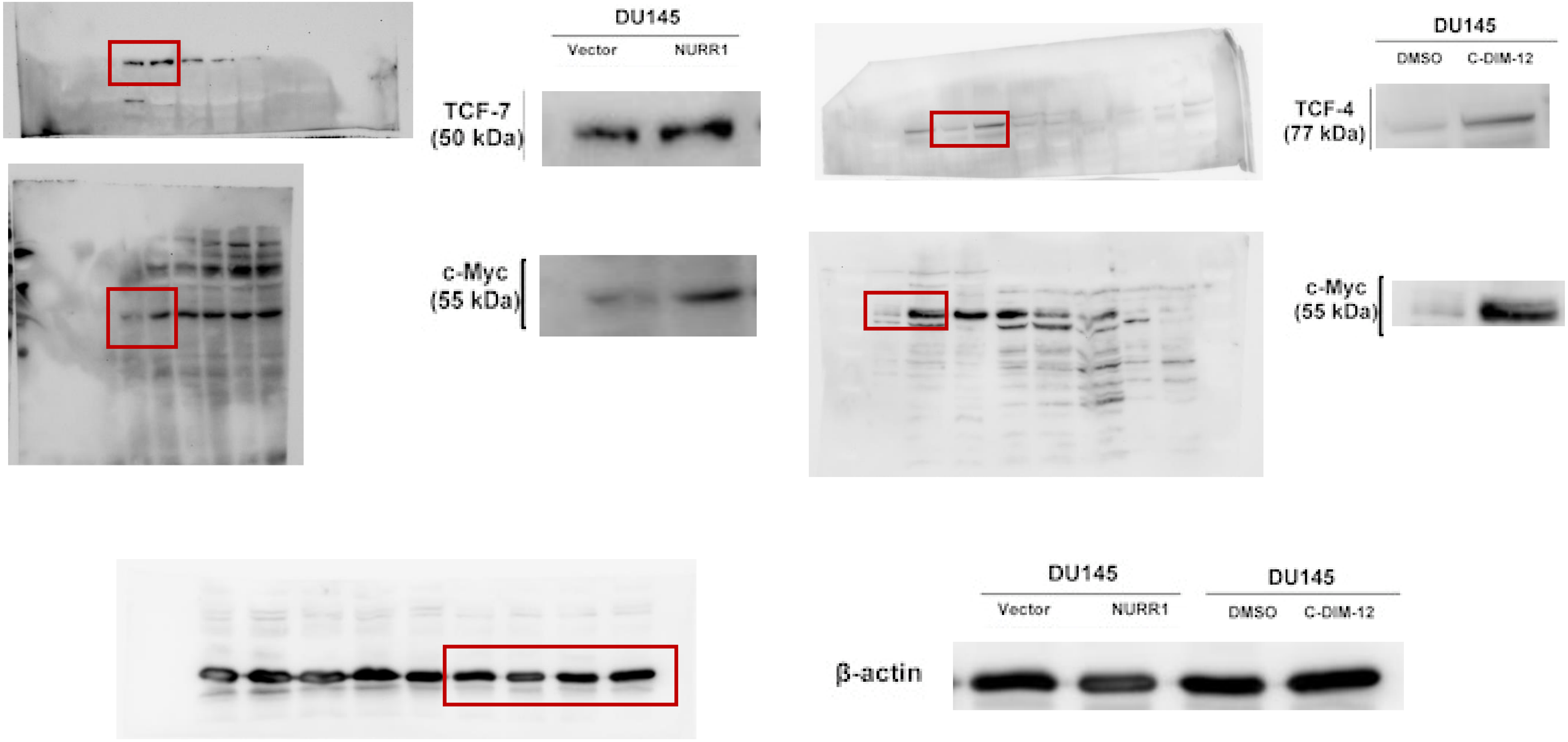
DU145
Vector
NURR1
TCF-7
(50 kDa)
c-Myc
(55 kDa)
DU145
DMSO
C-DIM-12
TCF-4
(77 kDa)
c-Myc
(55 kDa)
DU145
Vector
NURR1
DU145
DMSO
C-DIM-12
β-actin

Figure 3G DU 145

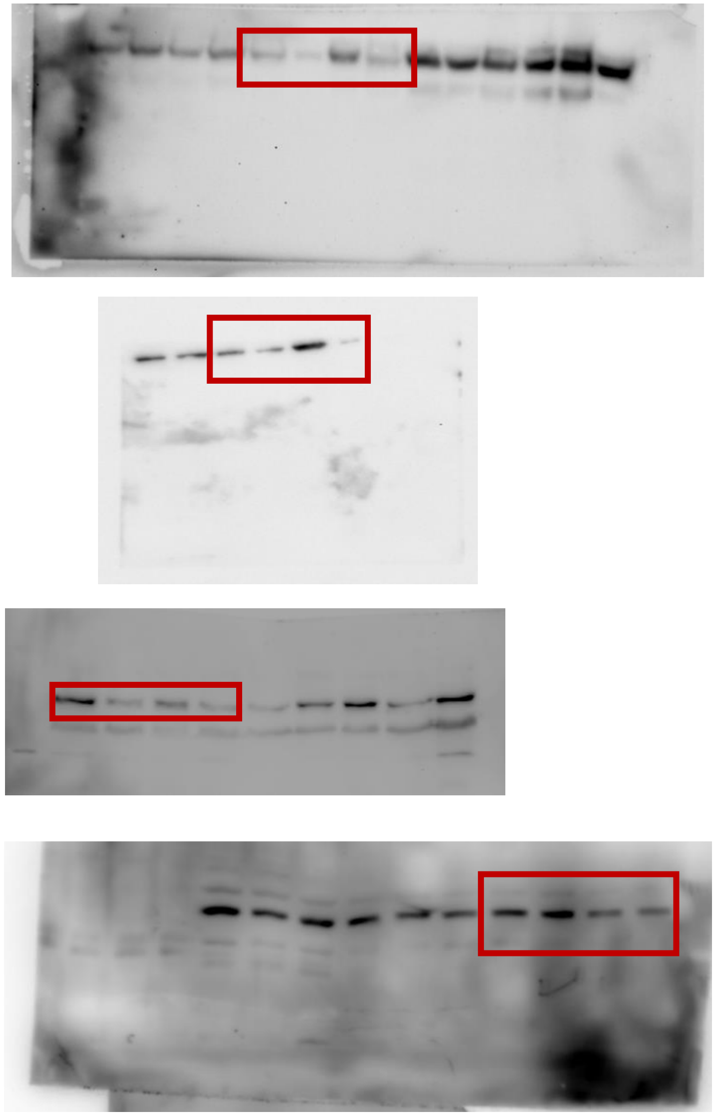

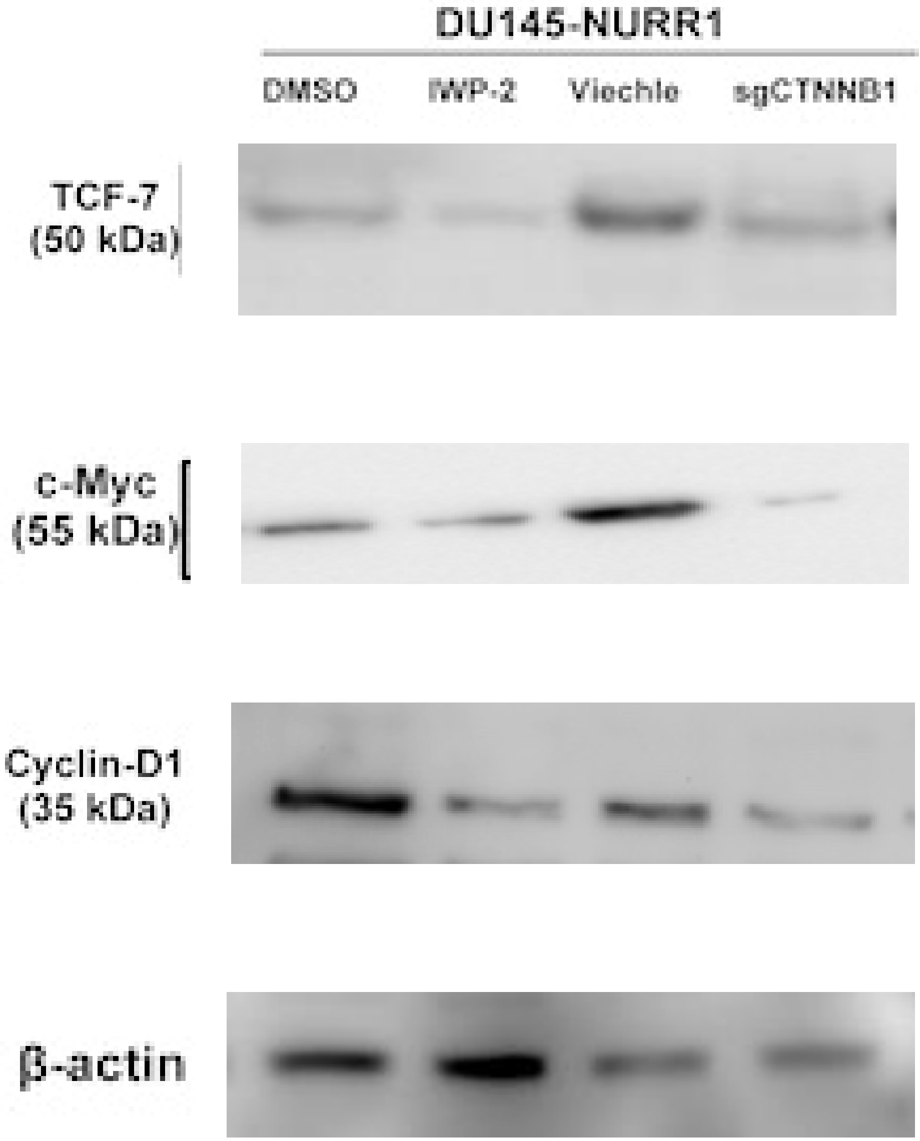

Figure 3G 22Rv1

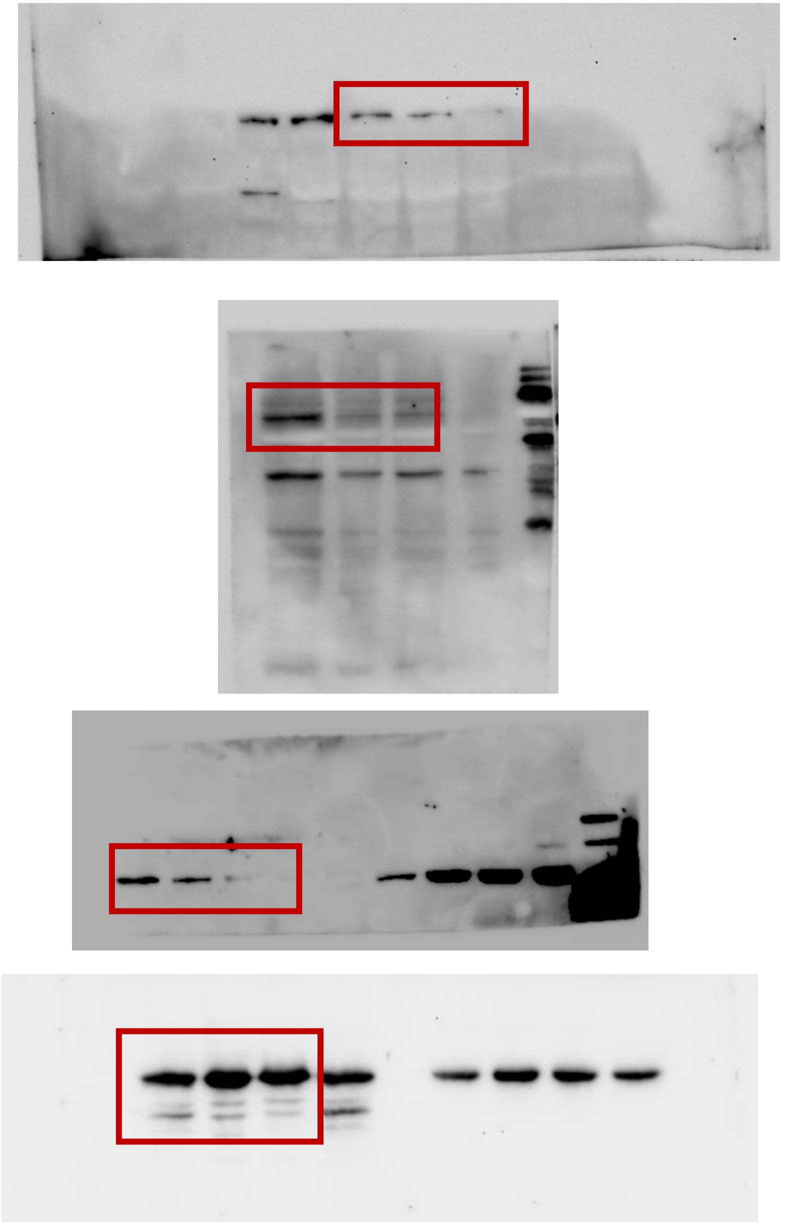

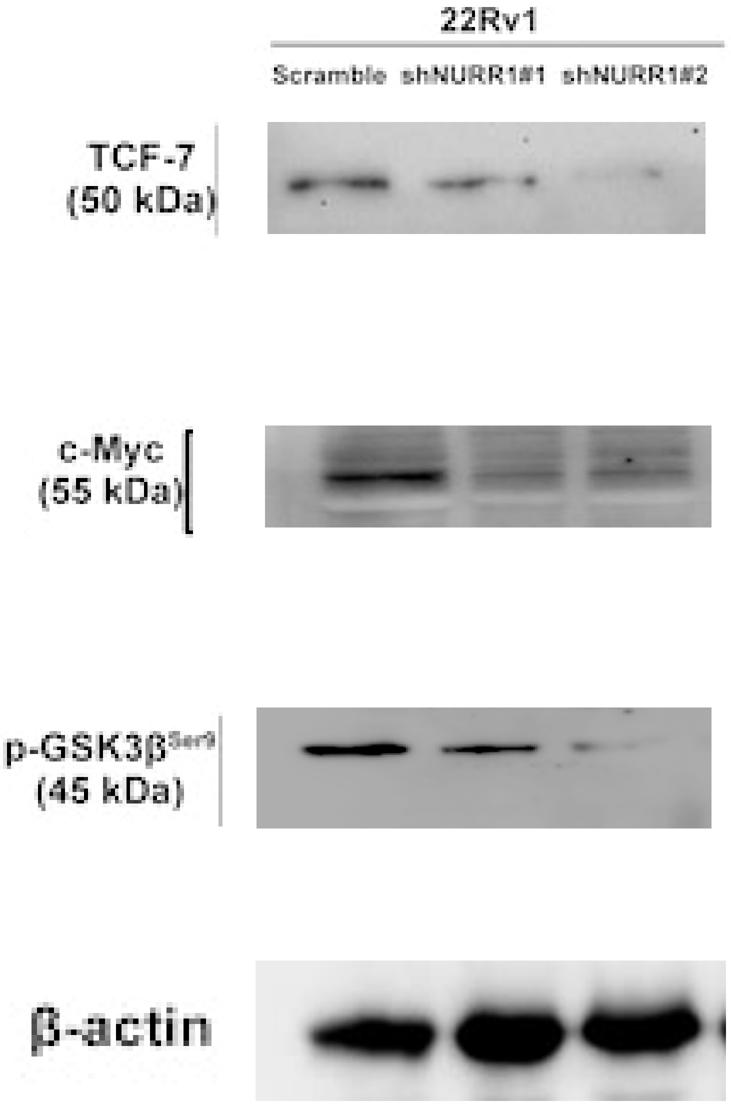

Figure 4E LNCaP

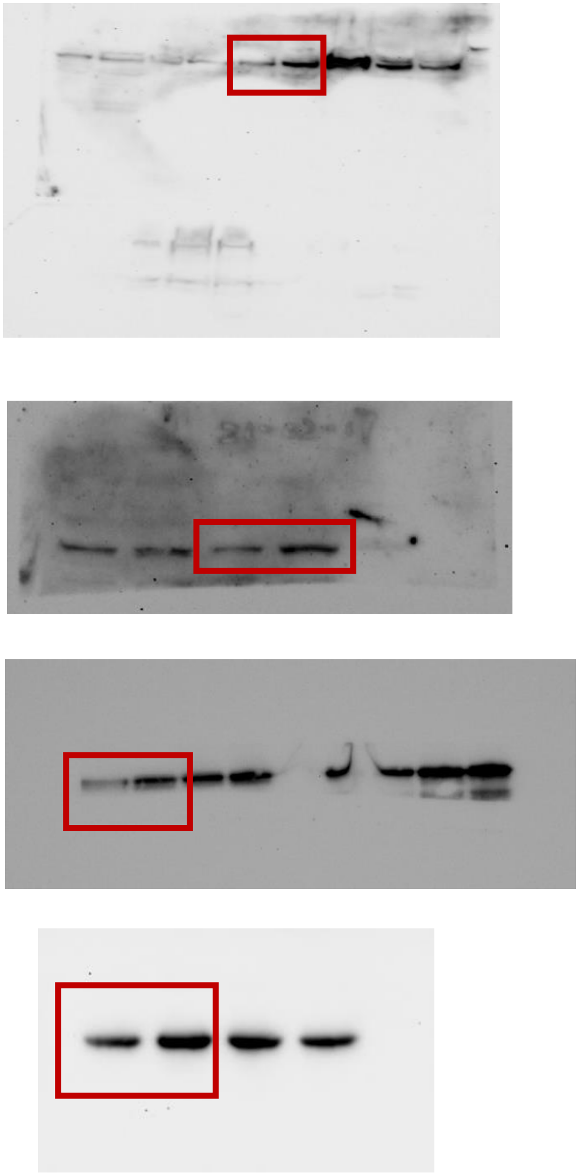

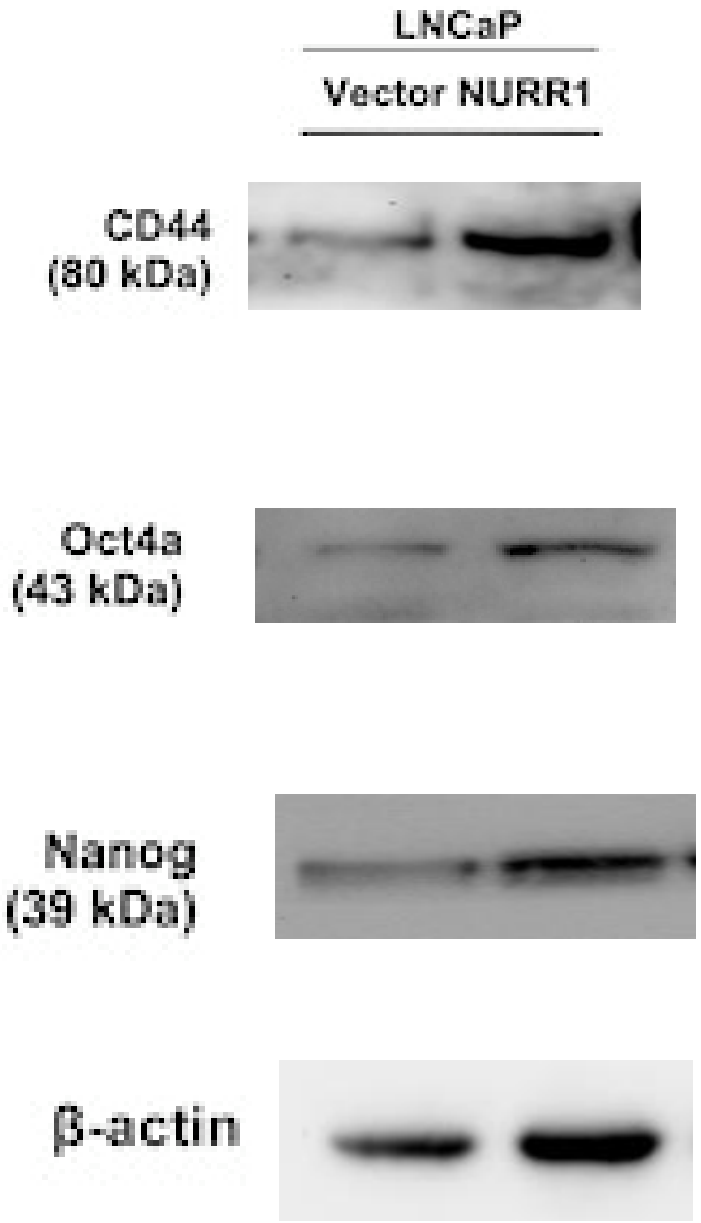

Figure 4E VCaP

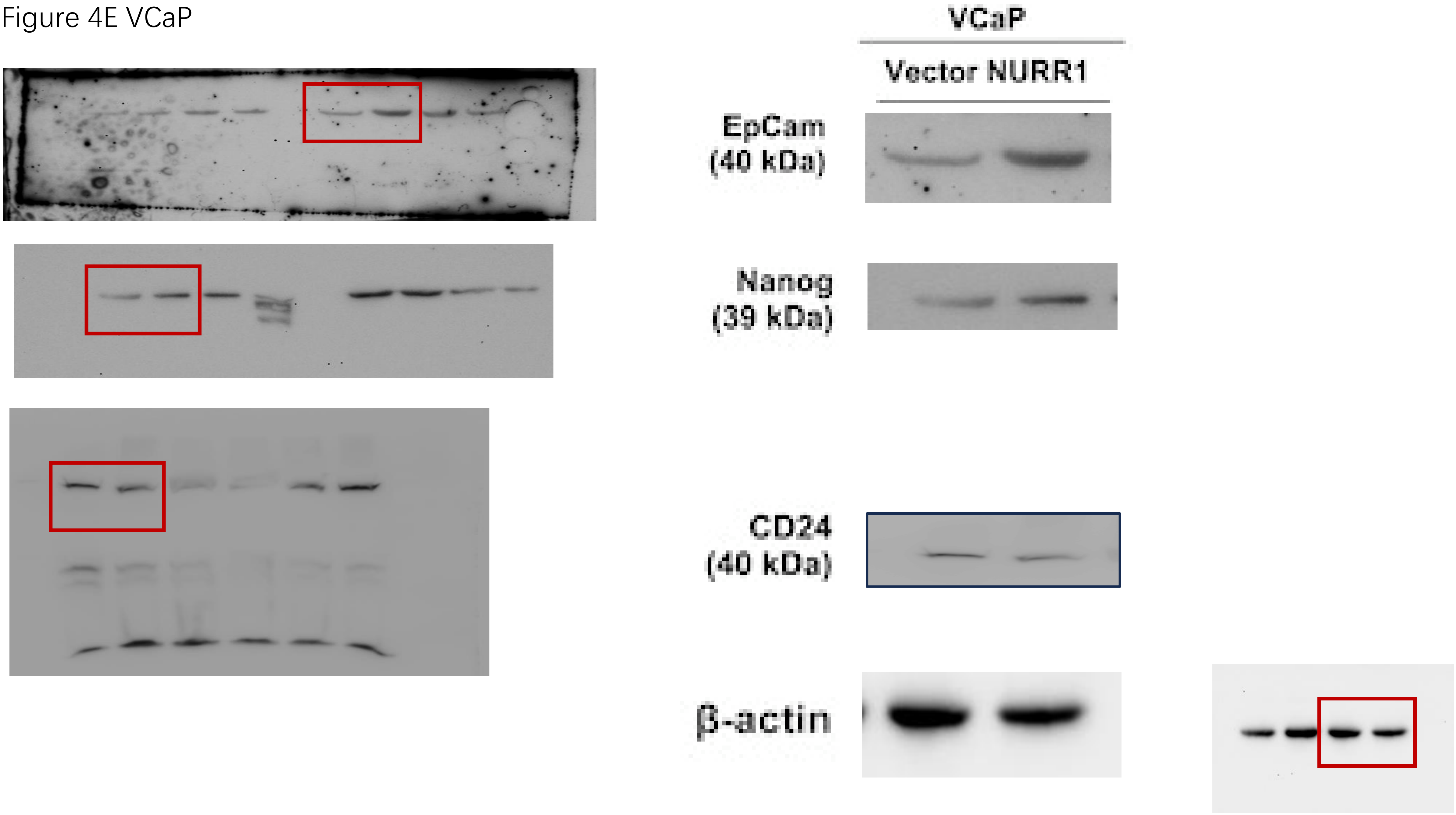

Figure 4F DU 145-OE

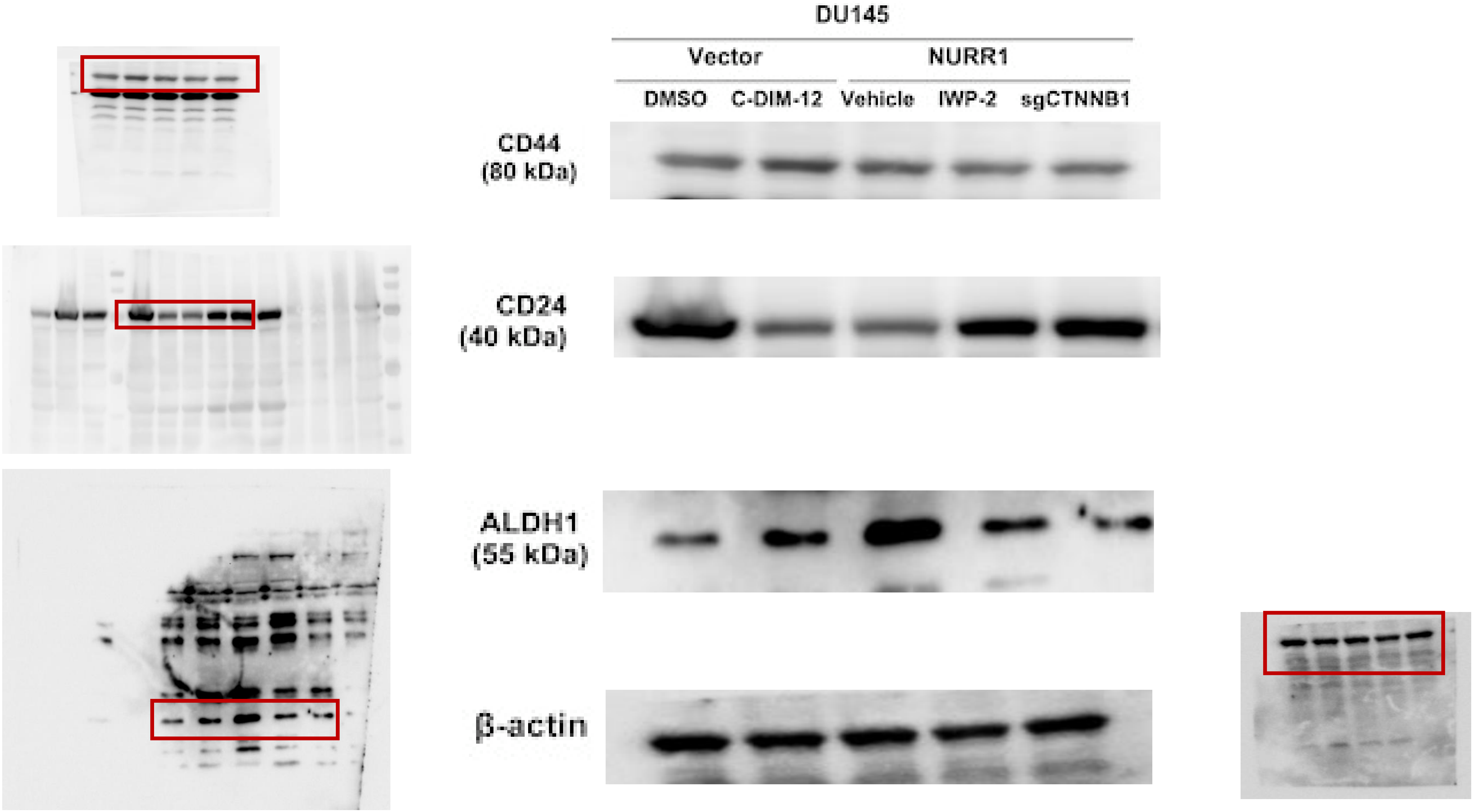

Figure 4F DU 145-KD

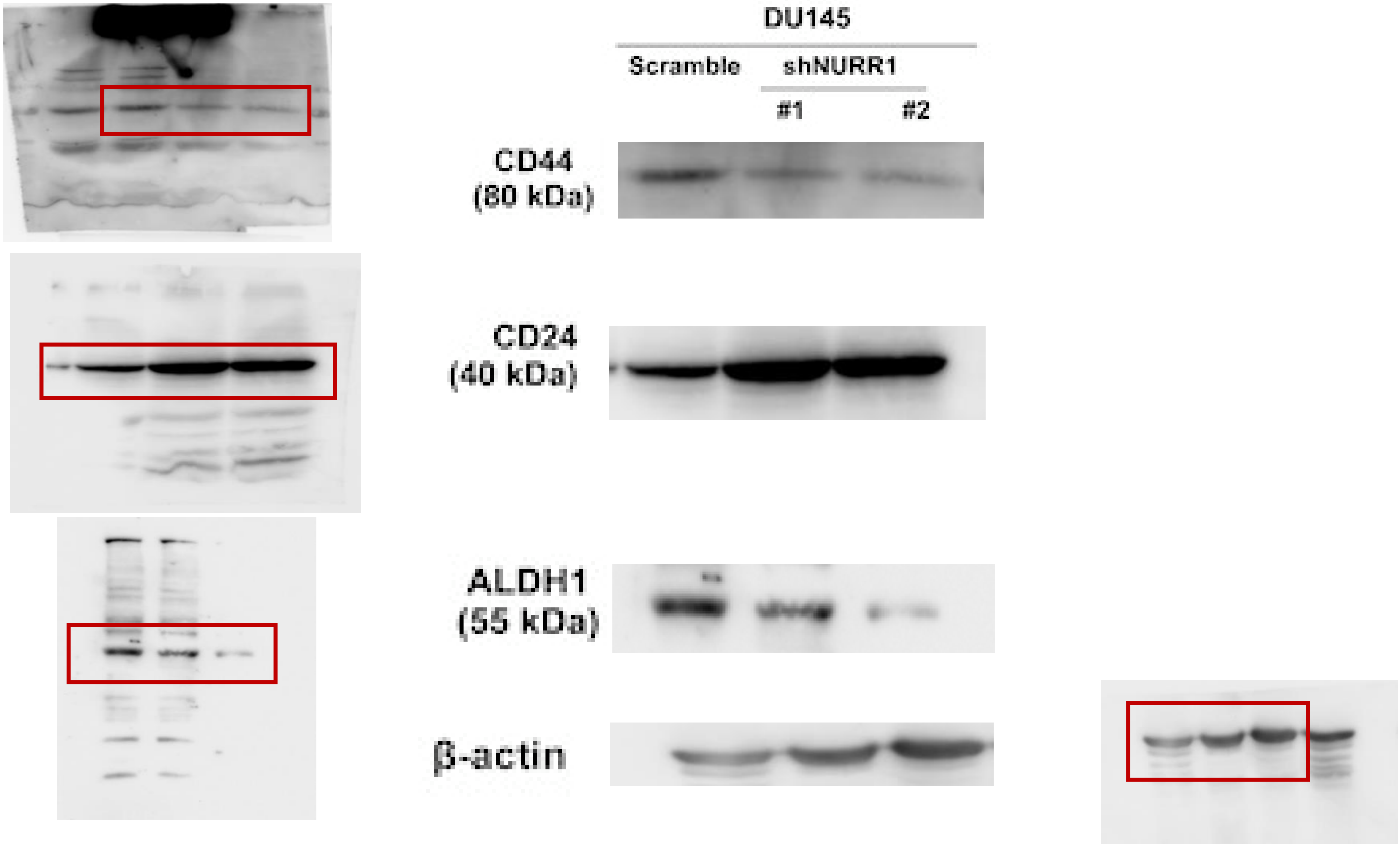

Figure 5C NURR1, β-catenin, TCF-4

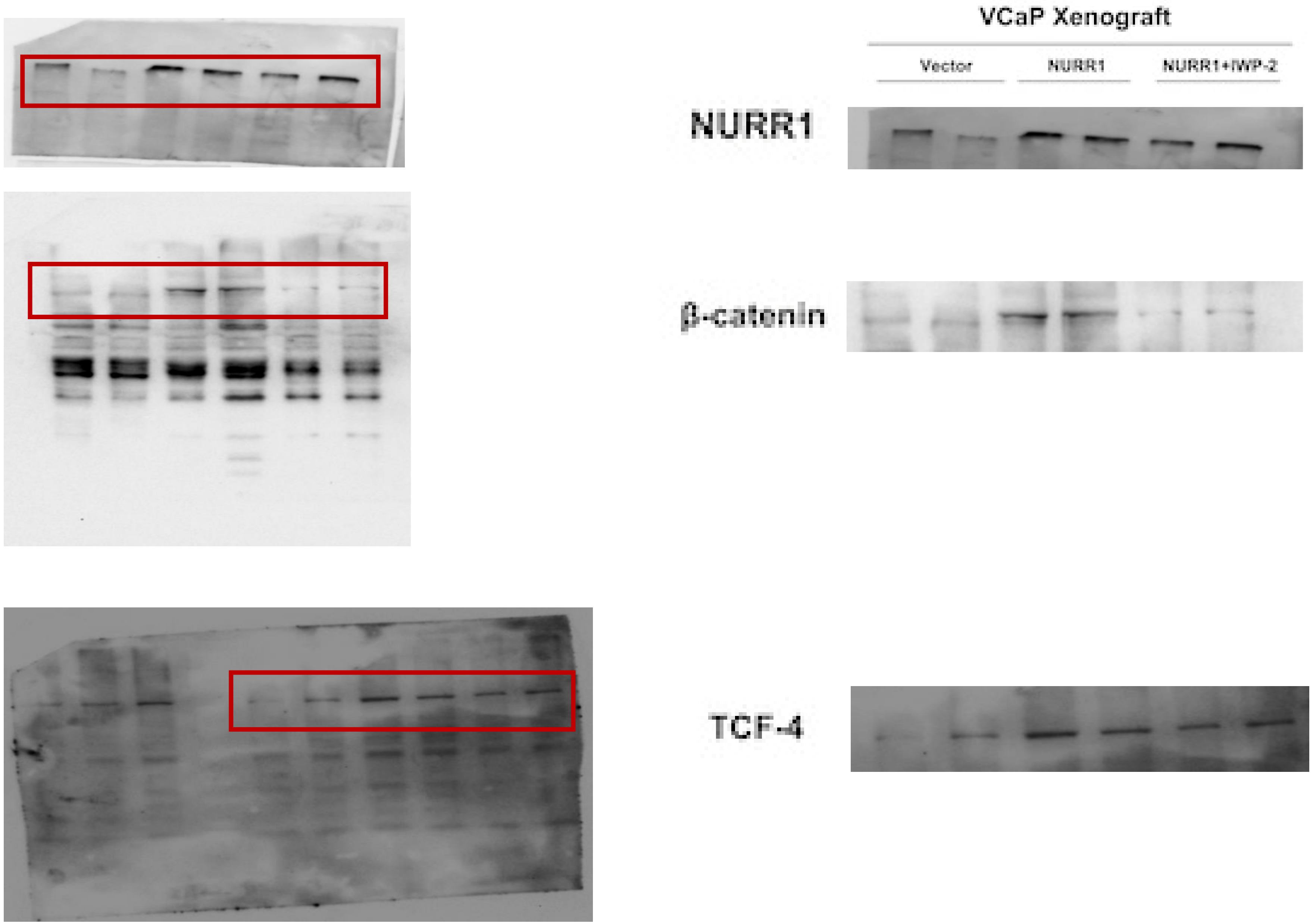

Figure 5C TCF-7, Cyclin D1, E-Cad, Vimentin

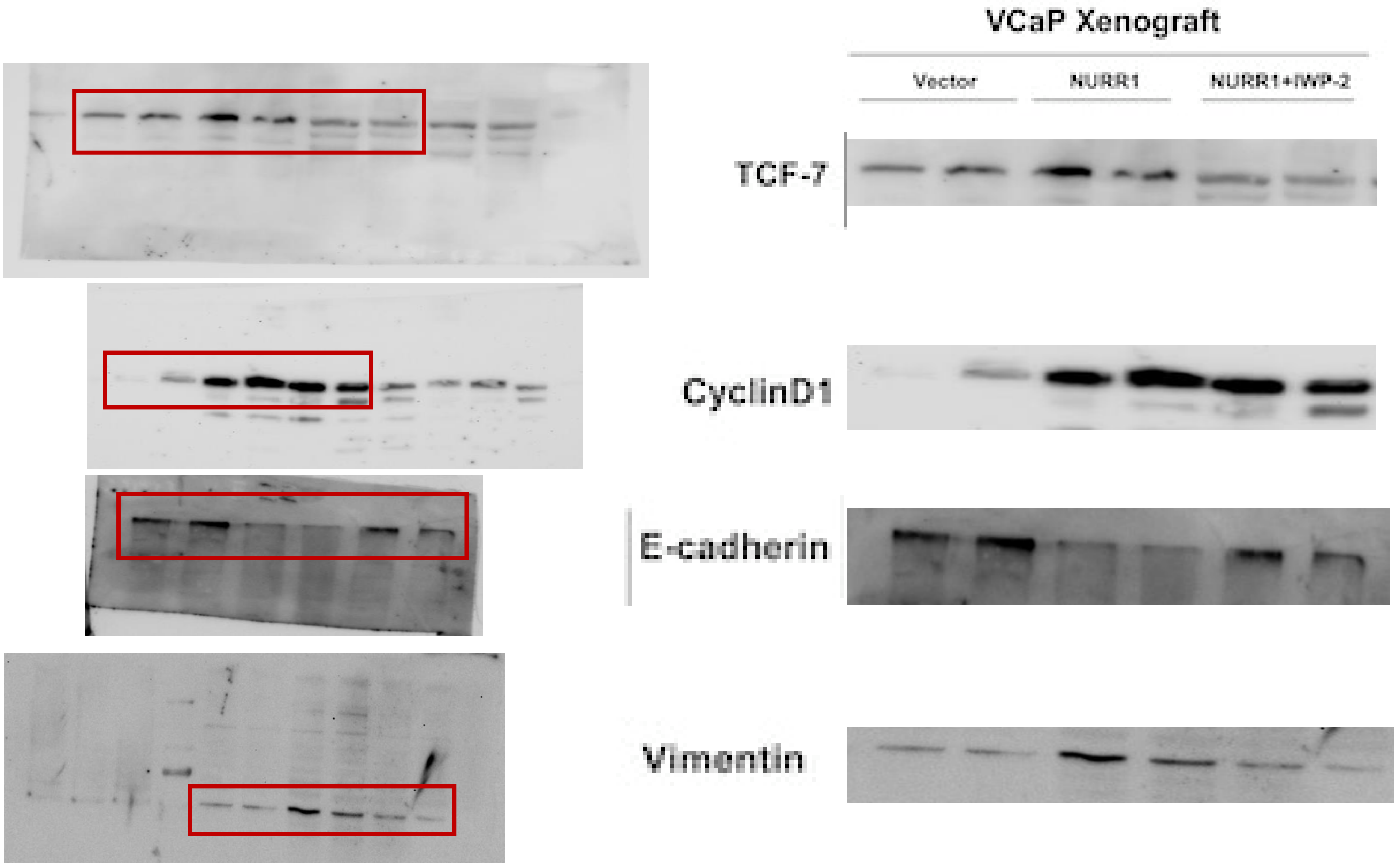

Figure 5C CD44, CD24, Oct4a, β-actin

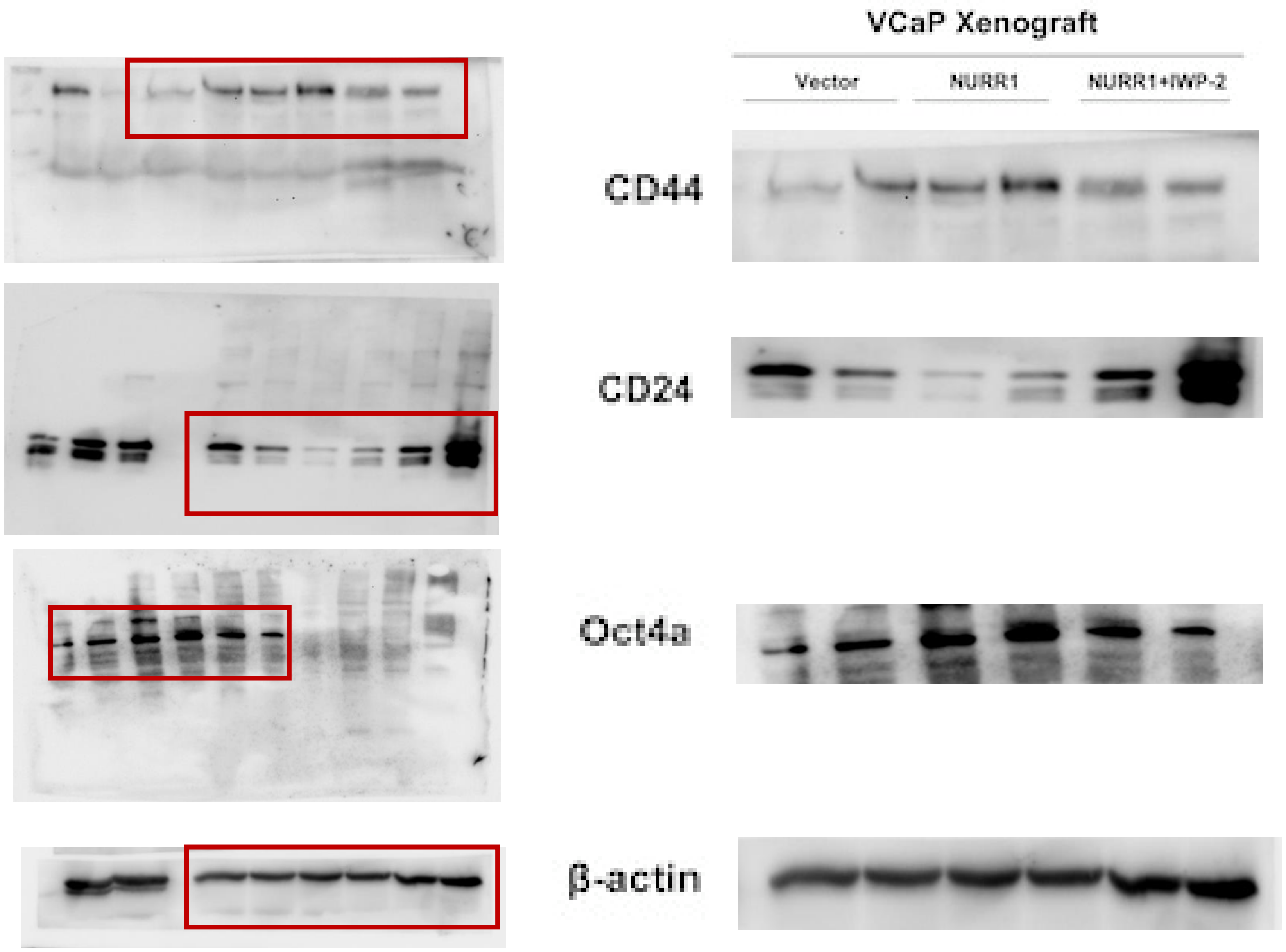

Figure 6E

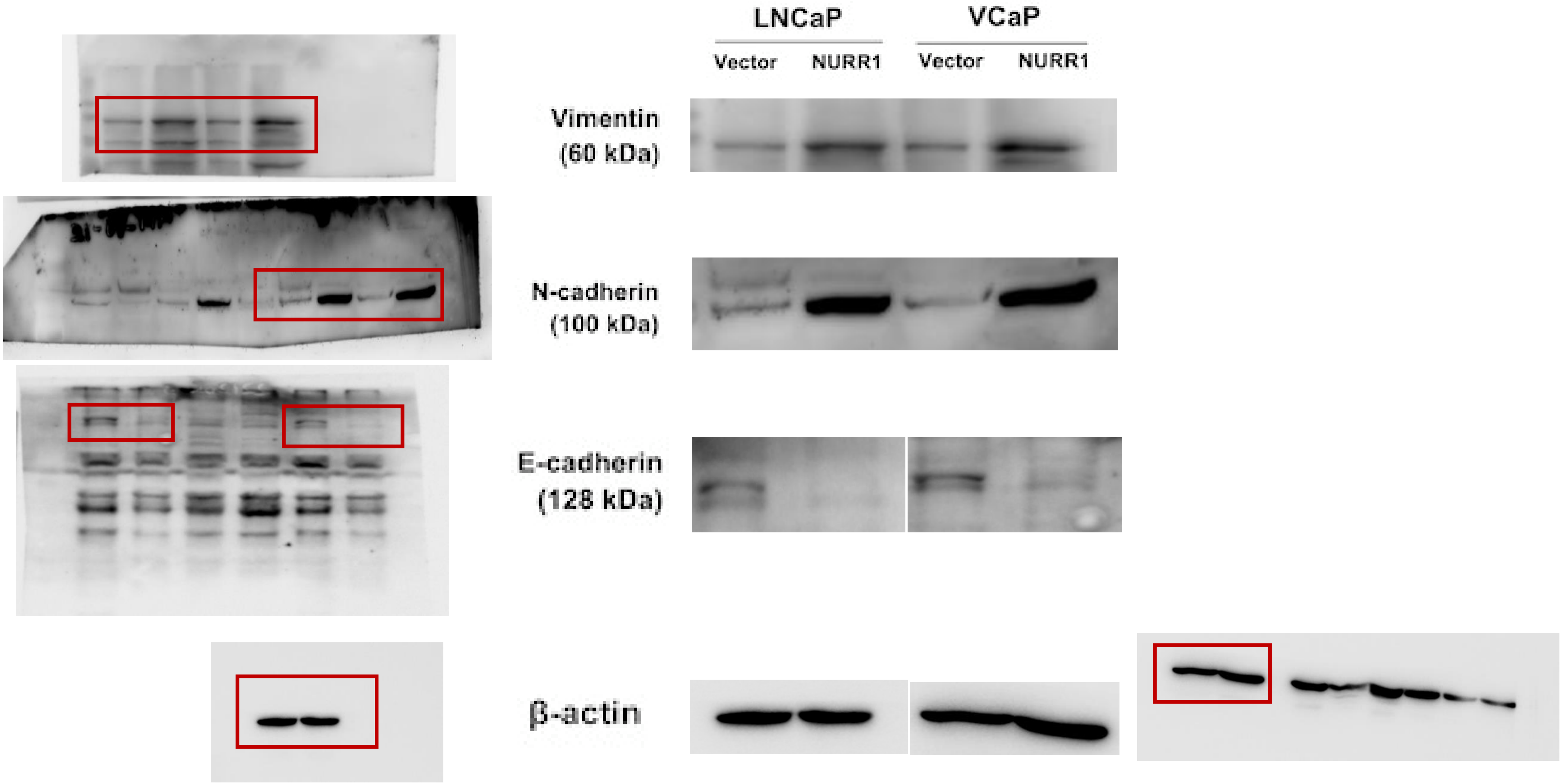

LNCaP
VCaP
Vector
NURR1
Vector
NURR1
Vimentin
(60 kDa)
N-cadherin
(100 kDa)
E-cadherin
(128 kDa)
β-actin

Figure 6F PC-3

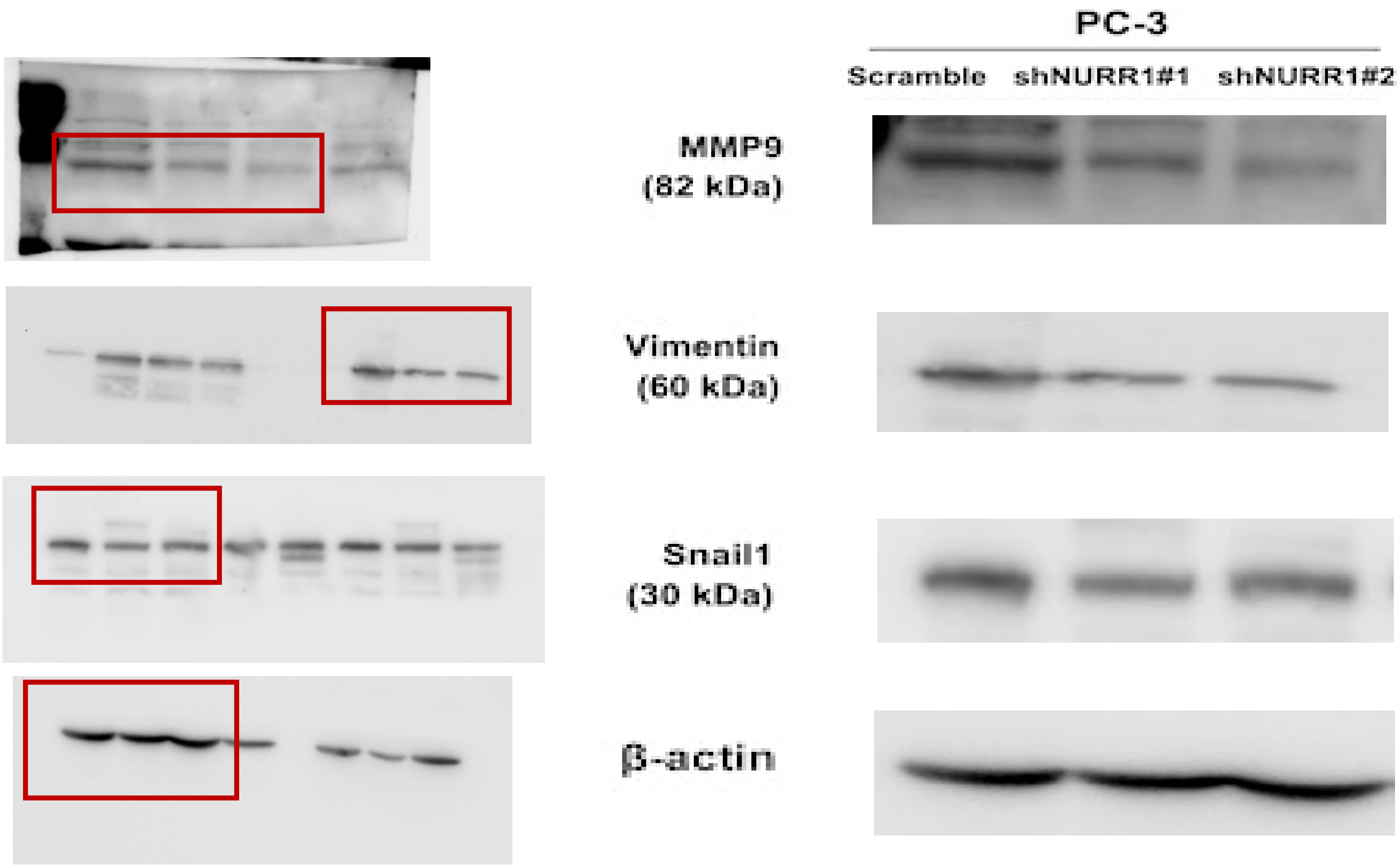

Figure 6F DU 145

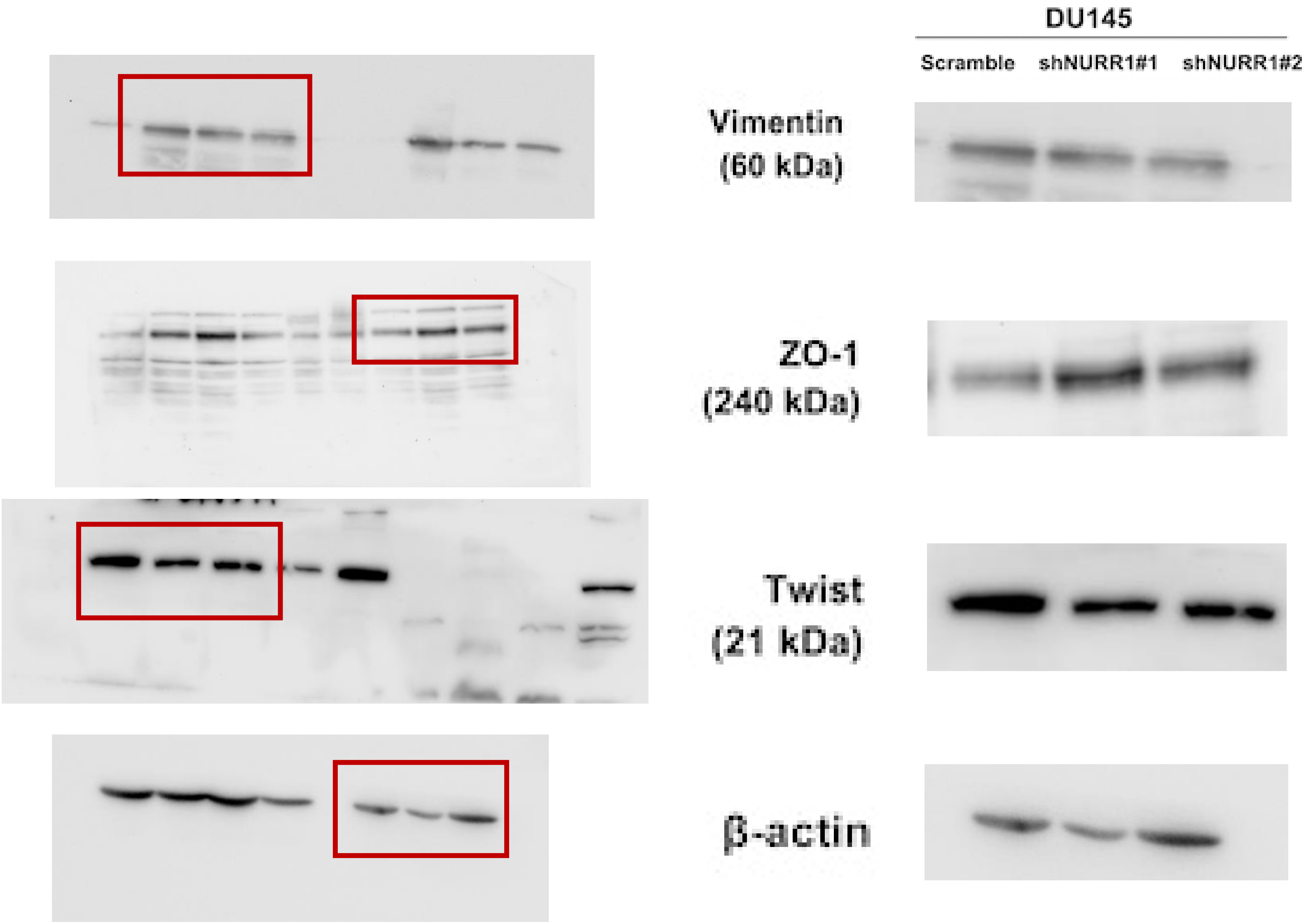

Figure 6G

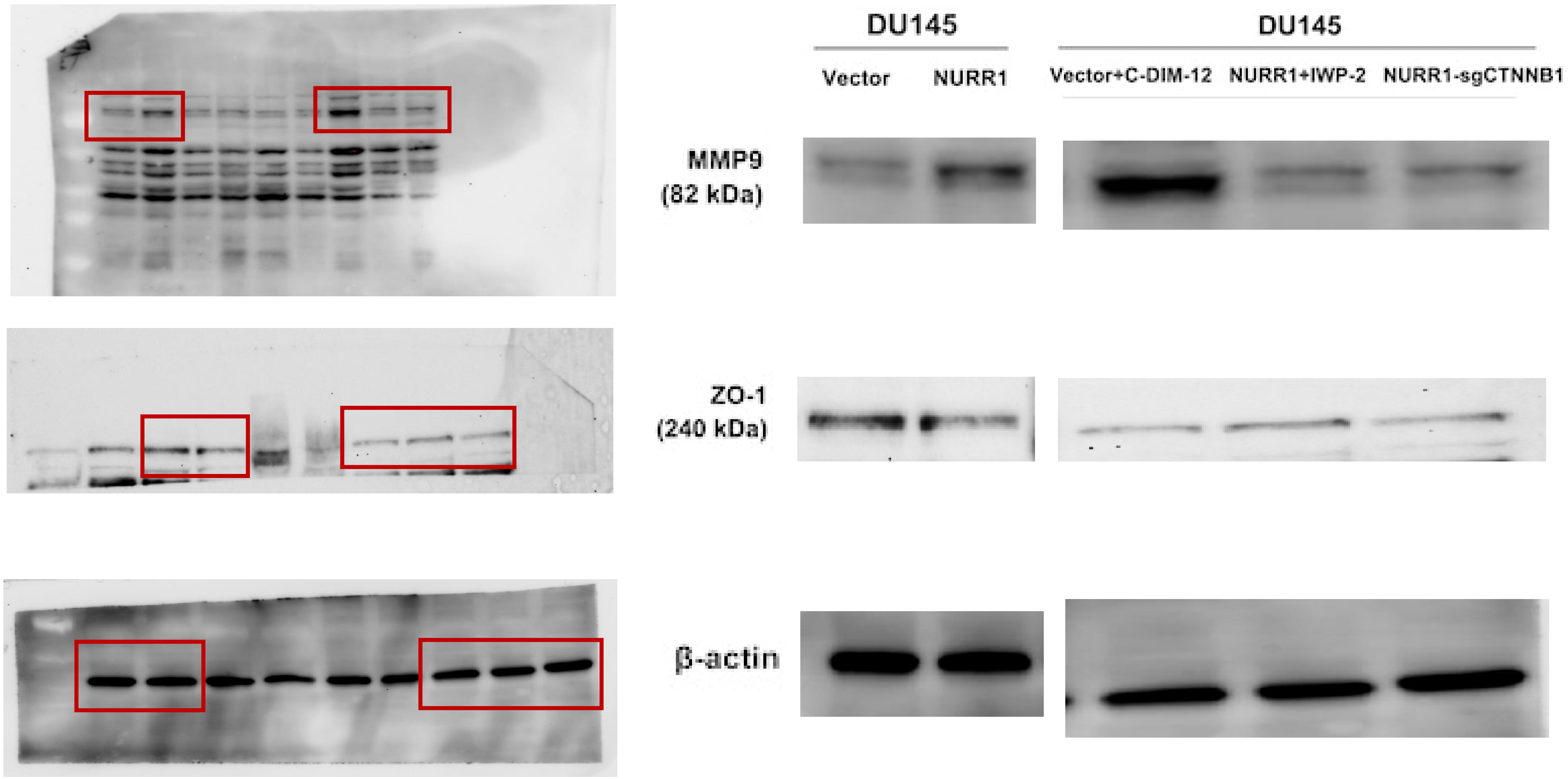
DU145
DU145
Vector
NURR1
Vector+C-DIM-12
NURR1+IWP-2
NURR1-sgCTNNB1
MMP9
(82 kDa)
ZO-1
(240 kDa)
β-actin
